# Population Dynamics and Parasite Load of a Foraminifer on Its Antarctic Scallop Host with Their Carbonate Biomass Contributions

**DOI:** 10.1371/journal.pone.0132534

**Published:** 2015-07-17

**Authors:** Leanne G. Hancock, Sally E. Walker, Alberto Pérez-Huerta, Samuel S. Bowser

**Affiliations:** 1 Department of Geology, University of Georgia, Athens, Georgia, United States of America; 2 Department of Geological Sciences, University of Alabama, Tuscaloosa, Alabama, United States of America; 3 Wadsworth Center, New York State Department of Health, Albany, New York, United States of America; University of California, UNITED STATES

## Abstract

We studied the population dynamics and parasite load of the foraminifer *Cibicides antarcticus* on its host the Antarctic scallop *Adamussium colbecki* from three localities differing by sea ice cover within western McMurdo Sound, Ross Sea, Antarctica: Explorers Cove, Bay of Sails and Herbertson Glacier. We also estimated CaCO_3_ biomass and annual production for both species. *Cibicides* populations varied by locality, valve type, and depth. Explorers Cove with multiannual sea ice had larger populations than the two annual sea ice localities, likely related to differences in nutrients. Populations were higher on *Adamussium* top valves, a surface that is elevated above the sediment. Depth did not affect *Cibicides* distributions except at Bay of Sails. *Cibicides* parasite load (the number of complete boreholes in *Adamussium* valves) varied by locality between 2% and 50%. For most localities the parasite load was < 20%, contrary to a previous report that ~50% of *Cibicides* were parasitic. The highest and lowest parasite load occurred at annual sea ice localities, suggesting that sea ice condition is not important. Rather, the number of adults that are parasitic could account for these differences. *Cibicides* bioerosion traces were categorized into four ontogenetic stages, ranging from newly attached recruits to parasitic adults. These traces provide an excellent proxy for population structure, revealing that Explorers Cove had a younger population than Bay of Sails. Both species are important producers of CaCO_3_. *Cibicides* CaCO_3_ biomass averaged 47-73 kg ha^-1^ and *Adamussium* averaged 4987-6806 kg ha^-1^ by locality. Annual production rates were much higher. Moreover, *Cibicides* represents 1.0-2.3% of the total host-parasite CaCO_3_ biomass. Despite living in the coldest waters on Earth, these species can contribute a substantial amount of CaCO_3_ to the Ross Sea and need to be incorporated into food webs, ecosystem models, and carbonate budgets for Antarctica.

## Introduction

Parasites are emerging as important ecological agents for structuring plant and animal communities, signifying the vitality or degradation of ecosystems [1–3]. Parasitic biomass often exceeds that of top predators in marine systems, affecting ecosystem function [4]. Despite their importance, the majority of food web topologies exclude parasites [5–6]. Indeed, the most recent food-web topology for Antarctic ecosystems does not include parasites or the ubiquitous foraminifera [7].

Foraminifera are diverse and abundant in marine ecosystems [8]. They enter into marine food webs at multiple trophic levels, including suspension feeding, grazing, predation, and parasitism [9–15]. Importantly, foraminifera exhibit multiple trophic modes [13]. Thereby, a single species enters into a food web at multiple levels, with implications for ecosystem function. Of these trophic modes, parasitism is the least well understood.

We examined the relationship between the parasitic foraminifer *Cibicides antarcticus* and its host, the Antarctic scallop *Adamussium colbecki*, which live in the coldest waters on Earth near the freezing point of seawater *(*-1.97 °C). Both species are circum-Antarctic in distribution. Epibenthic *Adamussium* is considered a major ecosystem engineer because of its large population size, often covering 100% of the seafloor with densities up to 90 m^-2^ in some locations [16–20].

*Cibicides antarcticus* (formerly *C*. *refulgens* in Antarctica) is a facultative parasite. It bores a hole through *Adamussium* valves with its pseudopods and assimilates ^14^C-labeled amino acids from the extrapallial cavity [13, 20]. This cavity, which is located between the shell of the mollusk and the mantle, contains fluid that is compositionally different from that of seawater, and is often enriched in calcium ions and amino acids [13–14]. Parasitism is thought to occur in 50% of *Cibicides* populations at Explorers Cove, based on observations from five *Adamussium* valves [13]. However, a systematic examination of *C*. *antarcticus* populations and parasitism using a larger sample size of *Adamussium* from different Antarctic localities has not been conducted. If *Cibicides* also suspension feeds and grazes on diatoms [13], it would be important to document the contribution of parasitism to its trophic behavior.

We first examined living *Cibicides* populations and their bioerosion traces on *Adamussium* across three Antarctic marine localities from western McMurdo Sound in the Ross Sea: Explorers Cove, Bay of Sails and Herbertson Glacier. Explorers Cove is well known for its foraminifera [21–25]. Unlike the other localities, it has multiannual sea ice with partial melt outs since 1991 [26]. It is also situated at the mouth of the Taylor Dry Valley, part of the largest ice-free region in Antarctica [27]. Bay of Sails and Herbertson Glacier experience annual sea ice and are adjacent to glaciated terrane [26, SSB and SEW personal observations]. Microalgae and microbes associated with sea ice are a major food resource for Arctic and Antarctic organisms [28–29]. Foraminifera populations are higher at Explorers Cove than Bay of Sails, and this difference could be related to sea ice algae [26]. If so, then parasitism should be rare at Explorers Cove because suspension feeding and grazing would be more important trophic strategies.

*Cibicides antarcticus* etches permanent traces into *Adamussium* shells [13]. We categorized these traces into four ontogenetic stages ranging from new recruits to parasitic adults. These stages were then used to examine population structure, spatial distribution, and parasitism in *Cibicides*. Within this framework, traces can be used in modern and fossil communities as an important life history proxy for *Cibicides* when it is no longer attached to the shell. Moreover, because *C*. *antarcticus* and *A*. *colbecki* have a fossil record extending to the Pliocene in Antarctica [30–31], these traces provide an evolutionary archive for the development of the host-parasite relationship.

Lastly, we estimated the CaCO_3_ biomass produced by the host-parasite relationship, as biomass is a long-established measure for ecosystem energetics [4]. In deep-sea communities, foraminifera account for up to 50% of the biomass derived from eukaryotic organisms [32]. In tropical reefs, foraminifera may contribute up to 43 million tons of CaCO_3_ per year (3.90 x10^10^ kg yr^-1^), which has significant implications for CO_2_ production in the world’s oceans [33]. In particular, reef and planktonic foraminifera may contribute ~1.4 billion tons of CaCO_3_ a year (1.27 x 10^12^ kg yr^-1^) accounting for ~ 25% of oceanic carbonate production [34]. While reef and planktonic foraminifera are well studied, we know little about the role of polar foraminifera in contributing CaCO_3_ to the world’s oceans. In fact, CaCO_3_ estimates for polar regions are generally lacking from global CaCO_3_ budgets [35–36]. Recently, macrozoobenthic organisms were described as unimportant for Antarctic CaCO_3_ budgets [37]. However, that study did not include foraminifera or other epibenthic communities important for estimating CaCO_3_ production. Therefore, we also examined the CaCO_3_ biomass and annual production for *C*. *antarcticus* and its host *A*. *colbecki*.

## Materials and Methods

### Study locations

**Fig 1:**
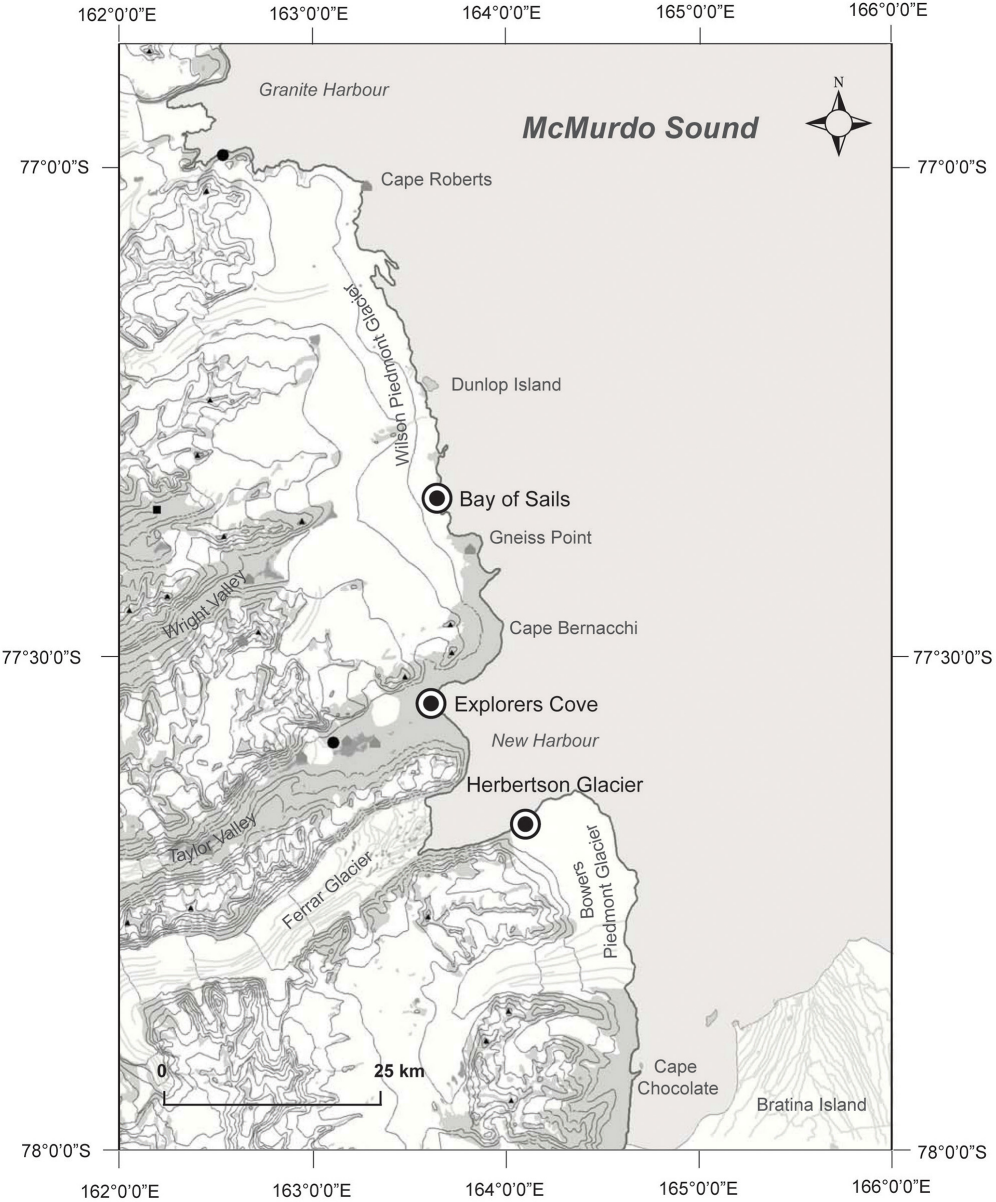
Localities in McMurdo Sound, Ross Sea, Antarctica. Map modified with permission from Antarctic New Zealand.

We examined *Cibicides* populations from three localities within McMurdo Sound, Ross Sea, Antarctica (Fig 1): Explorers Cove (EC, 77°34.259'S, 163°30.699'E), Bay of Sails (BOS, 77°21.911'S, 163°32.594'E) and Herbertson Glacier (HG, 77°41.724'S, 163°54.662'E). McMurdo Sound represents the southernmost extension of the Ross Sea [25]. These localities occur in western McMurdo Sound, a region characterized by oligotrophic, nutrient-depleted waters because the water mass that bathes this region has circulated under the Ross Ice Shelf [38–39]. Benthic and water column primary productivity is higher in eastern than western McMurdo Sound [40–41]. Diatom and organic carbon fluxes are up to two orders of magnitude greater in eastern McMurdo Sound [42–43]. As a consequence, the benthic community in western McMurdo Sound resembles the bathyal deep sea [43]. Dominant megafauna in this region include epibenthic *Adamussium*, ophiuroids, and echinoids [16, 38, 44].

Explorers Cove is a marine embayment located at the mouth of the Taylor Dry Valley [24]. It is characterized by gentle topography with rare glacial erratics [23]. Sediments are polymictic fine silty sands with a modal grain size of 125–300 μm [45]. Water currents are negligible except for tidal exchange, with estimates varying between <1 cm/sec to 2.6 cm/sec [46–47]. Sea ice has remained largely intact since 1993 with partial melt outs in 1999, 2002 and 2011 [26]. Iceberg disturbance has not occurred in this cove since 1981 [25]. During austral summer, glacially-derived freshwater flows into EC from Commonwealth and Wales deltas [13, 25]. At the time of collection, bottom water temperature was -1.97 °C with a salinity of 35–37 PSU and a pH of 7.6.

Bay of Sails is ~ 30 km north of EC and is located offshore of the Wilson Piedmont Glacier [26]. It is named for the persistent icebergs located offshore. Underwater topography is similar to EC, but with more glacial erratics [SSB personal observation]. Unlike EC, sea ice melts out every year at BOS [26]. Sediments are polymictic very fine sand with a modal grain size of 63–125 μm [26]. Water currents have not been measured and based on diving observations appear to be similar to EC [SSB personal observation]. Iceberg disturbance is likely high in this region, but has not been quantified. During austral summer BOS receives freshwater from the Wilson Piedmont Glacier [SEW personal observation]. At the time of collection, benthic seawater had a temperature of -1.97 °C, a salinity of 35.8 PSU and a pH ~ 7.9 [26].

The HG locality is located in shallow water just offshore of Herbertson Glacier in the Ferrar Glacier embayment. The topography is similar to BOS [SSB personal observation]. Icebergs were not observed in the region of Herbertson Glacier, but they can calve from the Ferrar Glacier [SEW personal observation]. The HG locality has annual sea ice cover and polymictic fine silty sands with a modal grain size of 125–250 μm (http://www.bowserlab.org). Bottom water had a temperature of -1.97 °C, a salinity of 35.6 PSU and a pH ~7.

Temperature, salinity and pH data were recorded by a YSI sonde deployed by divers at each depth. The sonde equilibrated for 10–15 minutes until stable readings were obtained. The pH probe is certified to -4 °C seawater and was calibrated with three pH standards (4.0, 7.0, and 10.0) prior to each dive. Salinity was calibrated using a 50mS cm^-1^ standardized solution from YSI International.

### Scallop collections

*Adamussium* were collected from two depths (9 m, 18 m) at each locality in November 2008. Scallops were haphazardly collected by divers (i.e., the nearest scallop bed that was closest to the dive site). Valves of similar size were targeted to control for surface area that could affect *Cibicides* abundance. Five top (left) and five bottom (right) valves were randomly chosen from each depth for a total of 20 valves per locality. Valve length, width, shell area, and weight were measured. Mean shell height was similar for EC and BOS (EC: n = 20, mean = 8.30 cm, SD = 0.30; BOS: n = 20, mean = 8.68 cm, SD = 0.38); valves from HG were slightly smaller (n = 20, mean = 7.11 cm, SD = 0.42).

### Living *Cibicides*

To determine if attached *Cibicides* were alive or dead on scallop valves, SSB tethered a live *Adamussium* with 118 *Cibicides* living on the top valve in 2005 at 21 m at EC. Video of tethered *Adamussium* was taken using a JVC Model VN-C30U steerable camera mounted in a custom-built underwater housing from Magee Scientific Company, Berkeley, CA. The top valve was retrieved a year later and fixed with 3% formalin, slightly decalcified, stained with 0.1% Rose Bengal, and examined using a Zeiss SMZ-2T stereomicroscope equipped with a SPOT model 29.2–1.3MP color camera. Attached *Cibicides* were counted from photographs and their spatial positions were compared to pre-deployment positions.

### *Cibicides* populations by locality, valve type, and depth

*Cibicides* populations were assessed three ways: 1) Attached *Cibicides* represent the living population, 2) bioerosion traces represent previous *Cibicides* populations that are no longer attached to the shell, and 3) attached *Cibicides* with traces left by previously attached *Cibicides* represents the total population that has accumulated over the lifespan of the scallop. We expected that *Cibicides* populations were not significantly different between localities, valve type (top and bottom valves) and depth.

*Cibicides* and their bioerosion traces were counted on *Adamussium* valves under a binocular/stereomicroscope at 200-750x. Selected *Cibicides* and traces were examined with a scanning electron microscope (SEM). To determine if *Cibicides* populations increased with increasing scallop area, attached *Cibicides* data were analyzed using a generalized linear model (GLM) with quasipoisson for over-dispersed count data [48]. Welch two-sample *t*-tests and multiple sample chi-squared tests were run with *Cibicides* and traces separately and then pooled to ascertain significant differences between localities, valve type, and depth. All statistics were performed in R at alpha = 0.05 unless otherwise specified [49].

### Parasite load

The parasite load represents the total number of complete boreholes made by *Cibicides* on each valve. We expected that the parasite load would not vary significantly between localities, valve type, and depth. We did expected that the parasite load would be higher on top valves than bottom valves because a small part of the bottom valve rests on the seafloor, decreasing surface area for foraminifera to settle. The mean number of *Cibicides* with and without complete boreholes for all localities were plotted with 95% CIs. Significant differences in the number of boreholes between depths and valve type were tested using Fisher Exact Tests (FETs).

### *Cibicides* population structure and spatial distribution

#### Population structure

**Fig 2:**
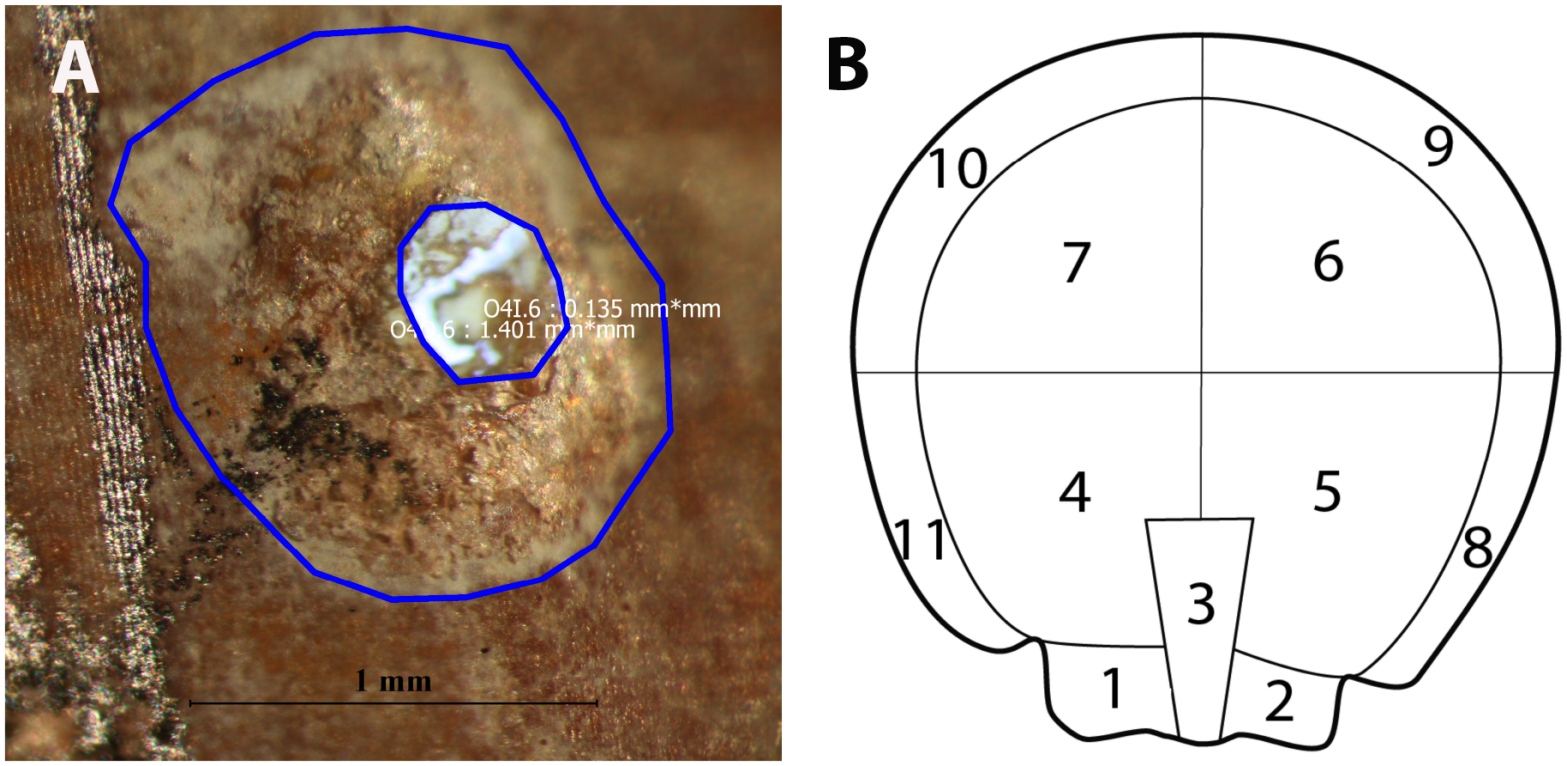
Trace Measurements and Sectors used to Examine *Cibicides* Population Structure on *Adamussium*. A. iSolution Lite image showing polygons that were used to measure exterior resting trace and interior borehole diameter. B. Sectors on *Adamussium* shell that were used to examine the spatial distribution of *Cibicides* bioerosion traces.

*Cibicides* population structure, from the initial attachment trace to the parasitic borehole, was assessed using bioerosion traces. We categorized the traces into ontogenetic stages (herein referred to as trace type) based on the size and morphology of the resting trace in relation to borehole development. We use “ontogenetic” because this term was used previously to examine young versus old bioerosion traces made by another parasitic foraminifera *Hyrrokkin sarcophaga*, although they did not categorize the traces into growth stages [50]. We then examined the spatial distribution of these trace types on *Adamussium* valves using six additional top valves from EC and BOS that were randomly selected from the initial scallop collection (n = 3 valves per locality). HG was omitted because the remaining shells were much smaller than those from EC and BOS. The outer resting trace and borehole diameter were measured in mm using the image analysis package iSolution Lite, IMT i-Solution Inc., Vancouver, Canada (Fig 2A). We used the outer resting trace because it represents the basal diameter of *C*. *antarcticus*. The borehole diameter will be discussed in a future paper. To determine if EC and BOS had significantly different trace types, Kruskal-Wallis ANOVAs (K-W tests) and post-hoc Wilcoxon tests (W tests) were used. The data were subset for the post-hoc tests yielding alpha = 0.008. We expected that trace-types would be similar between the localities.

#### Spatial distribution

Trace type spatial distribution was examined by dividing the shell surface into sectors (Fig 2B) (after [26, 51]). Sectors were selected based on shell topography because foraminifera are sensitive to microhabitats. *Cibicides* could settle in certain sectors to increase their ability to obtain nutrients from suspension feeding, grazing or parasitism. Sectors 1 and 2 are the auricles that were identified previously as an area with the greatest number of encrusting foraminifera [26]. Sector 3 is the umbo, the oldest part of the shell. Sectors 4–7 represent the center area of the valve, below which the mantle, muscle, gill, and gonadal tissue reside. Sectors 8–11 represent the outer edge of the scallop and the youngest shell surfaces. Densities for each trace type per sector were standardized to cm^2^ and were used to evaluate significant patterns in *Cibicides* spatial distributions. To determine the cut-off value for significantly higher or lower mean density of a particular trace type in a sector, one-sample *t*-tests were used to generate upper and lower 95% CIs. We expected that if *Cibicides* occurred randomly on *Adamussium* top valves, all trace types would have similar densities in each sector.

### CaCO_3_ biomass and annual production

*Cibicides* CaCO_3_ biomass was estimated in a series of steps. First, we weighed 579 individual *Cibicides* from two randomly selected EC top valves used in the previous population study. *Cibicides* were cleaned, dried at 70 °C for 24 hours, and weighed on a microbalance to the nearest mg to yield CaCO_3_ biomass. We assumed, like previous studies for foraminifera biomass, that their tests were primarily CaCO_3_ [33–34, 52]. Second, mean *Cibicides* CaCO_3_ biomass was binned into seven size classes of 0.10 mg increments and plotted with 95% CIs to determine if significant differences existed between size classes. Lastly, the mean for the entire sample was estimated. The mean provides a conservative estimate for CaCO_3_ biomass for foraminifera [33–34, 52].

We next estimated the CaCO_3_ biomass of *Adamussium*. First, we had to determine if their shells were mostly pure carbonate before using them for CaCO_3_ biomass estimates. Four top valves from EC were cleaned of epibionts, washed with DI water, dried, powdered and subjected to the Loss on Ignition method [53]. The shells yielded a mean organic carbon content of 0.010g C_org_ (SD = 0.001g; 0.03 wt% C_org_) indicating that *Adamussium* shells are primarily CaCO_3_. Valve weights were then used as a proxy for CaCO_3_ biomass.

CaCO_3_ biomass density was calculated in two steps. First, the total number of attached *Cibicides* on top valves was divided by the total shell area for each locality, yielding the mean number of *Cibicides* per cm^2^. Second, the mean number of *Cibicides* per cm^2^ was multiplied by the mean *Cibicides* biomass to provide a conservative estimate of CaCO_3_ biomass that was then converted to kg ha^-1^. The mean carbonate biomass density for *Adamussium* was calculated by dividing the total shell CaCO_3_ weight by the total shell area for each locality and converted to kg ha^-1^. Mean CaCO_3_ biomass density for both species was plotted with 95% CIs to determine if there were significant differences among localities. We then wanted to determine how much CaCO_3_ *Cibicides* contributes to the host-parasite relationship by examining its percent contribution [after 4]. This percent was calculated using the mean biomass estimate divided by the host + parasite biomass (in kg ha^-1^) for each locality.

We calculated the annual CaCO_3_ production for *Cibicides* and *Adamussium* because annual biomass production reveals ecosystem productivity [4]. To calculate yearly production, we had to determine the turnover rate of *Cibicides* and *Adamussium* and then convert their CaCO_3_ biomass to yearly production. We estimated that the turnover rate was two years for *Cibicides* and 20 years for *Adamussium*. *Cibicides* two-year turnover was based on experimental arrays deployed at EC in austral summer 2008 (SEW personal observation). The experimental arrays had clean *Adamussium* shell pieces encased within plankton-mesh bags. When the arrays were retrieved two years later (2010), adult *Cibicides* were attached to the shell pieces. Juveniles were also observed within a *Cibicides* test in 2008, indicating that they are released during austral summer. As a consequence, propagules may have entered the mesh bags in austral summer 2008, likely growing to their largest size within two years. Additionally, a survivorship analysis based on size classes generated from *Cibicides* biomass revealed that it has a Type I curve, exhibiting high juvenile survivorship with mortality increasing with age (S1 Fig). *Adamussium*’s lifespan is thought to be 20 years based on growth-band analysis and mark-recapture estimates [54–57]. Therefore, CaCO_3_ production per year was calculated based on the mean CaCO_3_ biomass per m^2^ for each species divided by their turnover rates and then converted to kg ha^-1^ yr^-1^.

Next, we wanted to compare CaCO_3_ biomass density across localities. This was calculated using the number of attached *Cibicides* and *Adamussium* at each locality and multiplying each species by its mean CaCO_3_ biomass. *Cibicides* and *Adamussium* mean biomass estimates per locality were plotted with 95% CIs to determine if there were significant differences among localities. We expected that scallop biomass would not differ among localities but *Cibicides* biomass would be variable because of variation in population size.

Lastly, we quantified the contribution that attached *Cibicides* makes to the total CaCO_3_ biomass produced by the species pair at each locality and on an annual basis. CaCO_3_ biomass contribution (%) was calculated by dividing *Cibicides* CaCO_3_ biomass by the total biomass from each species for each locality. Similarly, *Cibicides* annual CaCO_3_ biomass contribution (%) was calculated by dividing *Cibicides* yearly CaCO_3_ production by *Adamussium* + *Cibicides* yearly CaCO_3_ production. We expected that *Cibicides* would contribute less to the total host-parasite biomass and yearly carbonate production because of the differences in size between *Cibicides* and its host.

### Ethics Statement

This study was carried out under the auspices of the U.S. Antarctic Program of the National Science Foundation and their research permits.

## Results

### Living *Cibicides*

**Fig 3:**
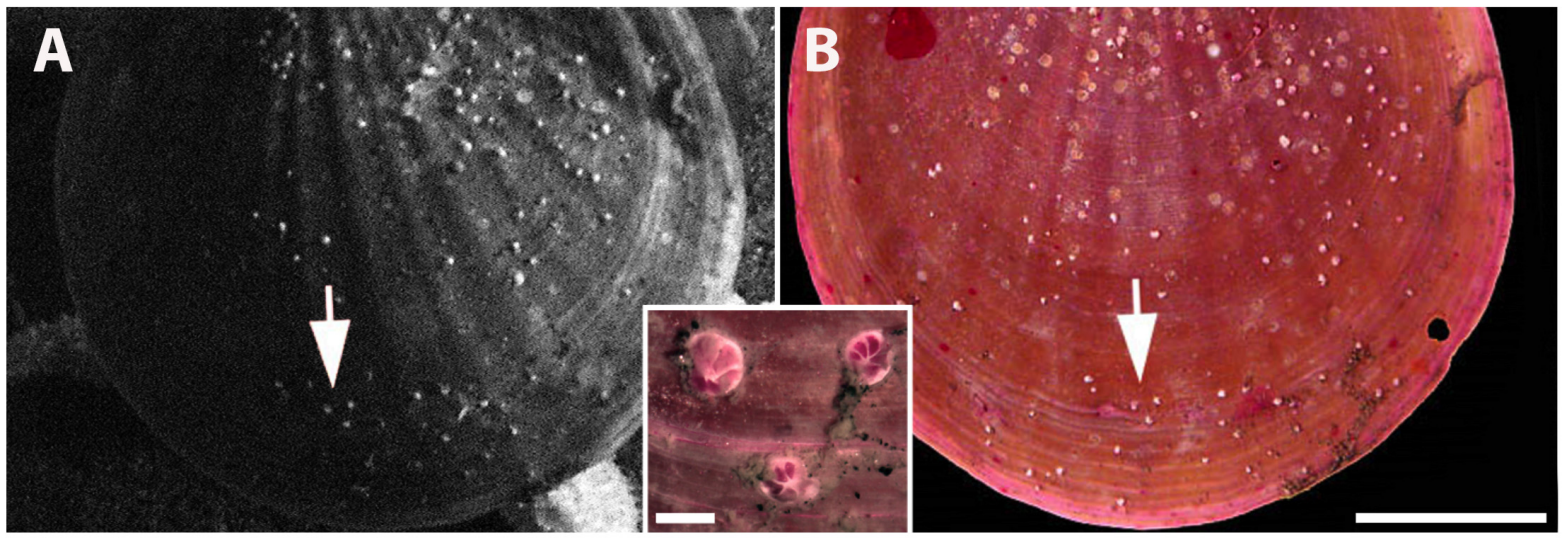
Experiment that Demonstrates Attached *Cibicides* are Alive. A. Live *Adamussium* deployed at EC in 2005 with attached *Cibicides*. Arms from a sea star can be seen behind the scallop, contributing to the scallop’s death. B. Top valve retrieved after one year and stained with Rose Bengal that stains living tissue pink. Inset: slightly decalcified *Cibicides* showing Rose Bengal-stained cytoplasm in living individuals. Arrow in A and B point to the three *Cibicides* that are figured in inset. Scale bar is 20 mm for A and B; inset scale bar is 1 mm.

Attached *Cibicides* are alive, and upon their death they are no longer attached to the shell. Video captured early in deployment showed a sea star feeding on the tethered *Adamussium* resulting in its death (Fig 3A). Despite the death of its host, *Cibicides* continued to live on the top valve until it was retrieved from the seafloor a year later. *Cibicides* cytoplasm was densely stained with Rose Bengal dye indicating that they were alive at the time of collection (Fig 3B, and inset Fig 3). A total of 118 *Cibicides* in the same position on the shell and an additional eight stained juveniles were present on the retrieved shell. This result indicates that attached *Cibicides* represent living individuals.

### *Cibicides* populations by locality, valve type, and depth

**Fig 4:**
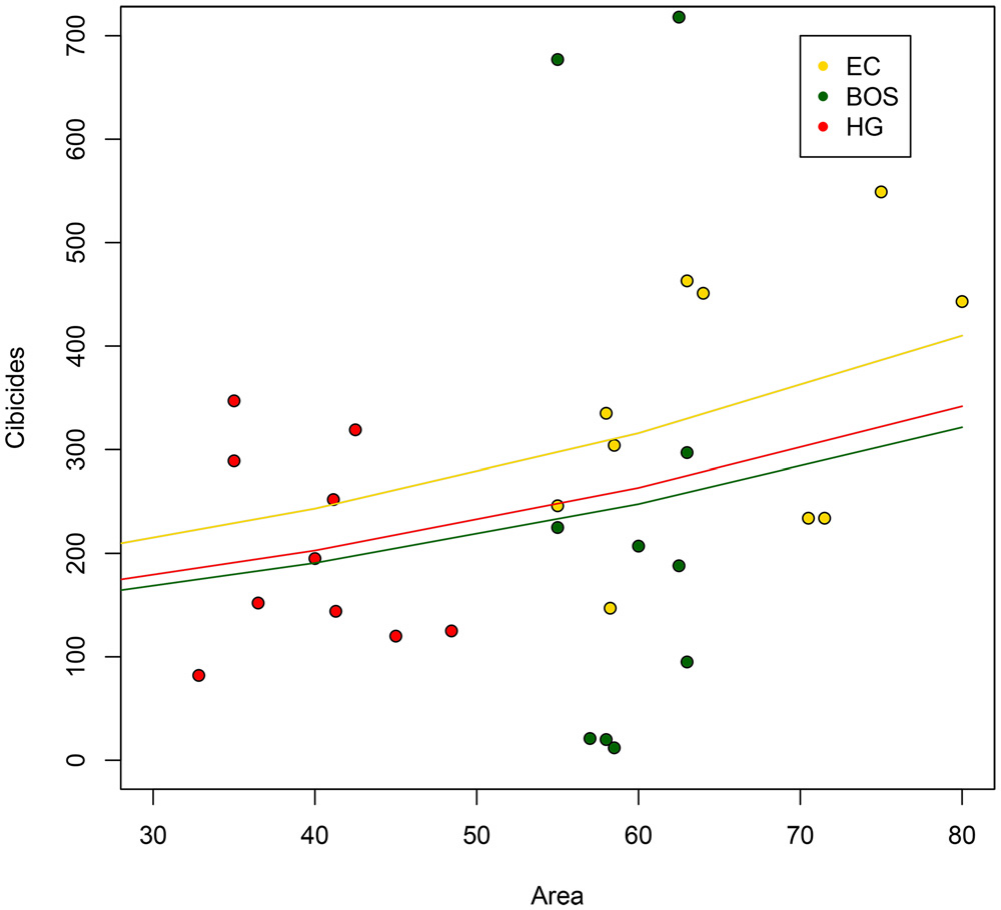
Generalized Linear Model of *Cibicides* Abundance by Shell Area. Localities: Explorers Cove (EC), Bay of Sails (BOS) and Herbertson Glacier (HG).

*Cibicides* had relatively high populations at all three localities (Table 1). BOS had the largest attached *Cibicides* population (39%, n = 1763 individuals) followed by EC (32%, n = 1461) and HG (29%, n = 1306). *Cibicides* mean densities, however, were higher at HG, averaging 33 cm^-2^ compared to 30 cm^-2^ for BOS and 23 cm^-2^ for EC. *Cibicides* bioerosion traces were more common at EC (58%, n = 2382) compared to BOS and HG, which had fewer traces (20%, n = 855 and 21%, n = 895, respectively). Mean density for traces was highest at EC (37 cm^-2^) followed by HG (23 cm^-2^) and BOS (15 cm^-2^) (Table 1). EC had a significantly larger total population representing 44% of all individuals (attached *Cibicides* + traces), while BOS had 30% and HG had 25% of the total population (chi-square = 756.87, df = 2, *p* < 0.0001). The abundance of attached *Cibicides* appeared to increase with shell area at all localities, but the trend was not significant because of high population variance among the shells (GLM with quasipoisson, *F* = 1.32, *p* = 0.35; Fig 4). Shell area was smaller at HG than the other localities, yet HG had a higher density of *Cibicides* supporting the GLM result.

**Table 1:** *Cibicides antarcticus* Populations by Antarctic Locality, Depth, and Valve Type. The number of attached *Cibicides* is reported in the numerator and the number of bioerosion traces is reported in the denominator (attached *Cibicides*/bioerosion traces). The frequency of occurrence within parentheses depicts the number of either attached *Cibicides* or bioerosion traces for each locality divided by the total pooled for all localities. The mean shell area for the Antarctic scallop *Adamussium colbecki* was used to determine the mean density of *Cibicides*.

Locality	Top	Bottom	Total	Mean Shell Area (cm^2^)	*Cibcides Mean* Density (cm^2^)
EC9	669/1060	45/184	714/1244	62.10	12/20
EC18	691/986	56/152	747/1138	68.65	11/17
EC Total	1360 (0.32)/2046 (0.57)	101(0.42)/336 (0.63)	1461(0.32)/ 2382 (0.58) = 3843 (0.44)	65.38	23/37
BOS9	198/138	11/28	209/166	59.80	4/3
BOS18	1474/650	80/39	1554/689	59.10	27/12
BOS Total	1672 (0.39)/788 (0.22)	91 (0.38)/67 (0.12)	1763 (0.39)/ 855 (0.20) = 2618 (0.30)	59.40	30/15
HG9	505/250	11/10	516/260	40.08	13/7
HG18	756/514	34/121	790/635	39.50	20/16
HG Total	1261 (0.29)/764 (0.21)	45 (0.19)/131 (0.24)	1306 (0.29)/ 895 (0.21) = 2201 (0.25)	39.79	33/23
Grand Total	4293/3598 = 7891 (0.91)	237/534 = 771 (0.09)	4530/4132 = 8662	—	—

EC, Explorers Cove; BOS, Bay of Sails; HG, Herbertson Glacier; Top and Bottom refer to top and bottom valves of *Adamussium colbecki*.

**Fig 5:**
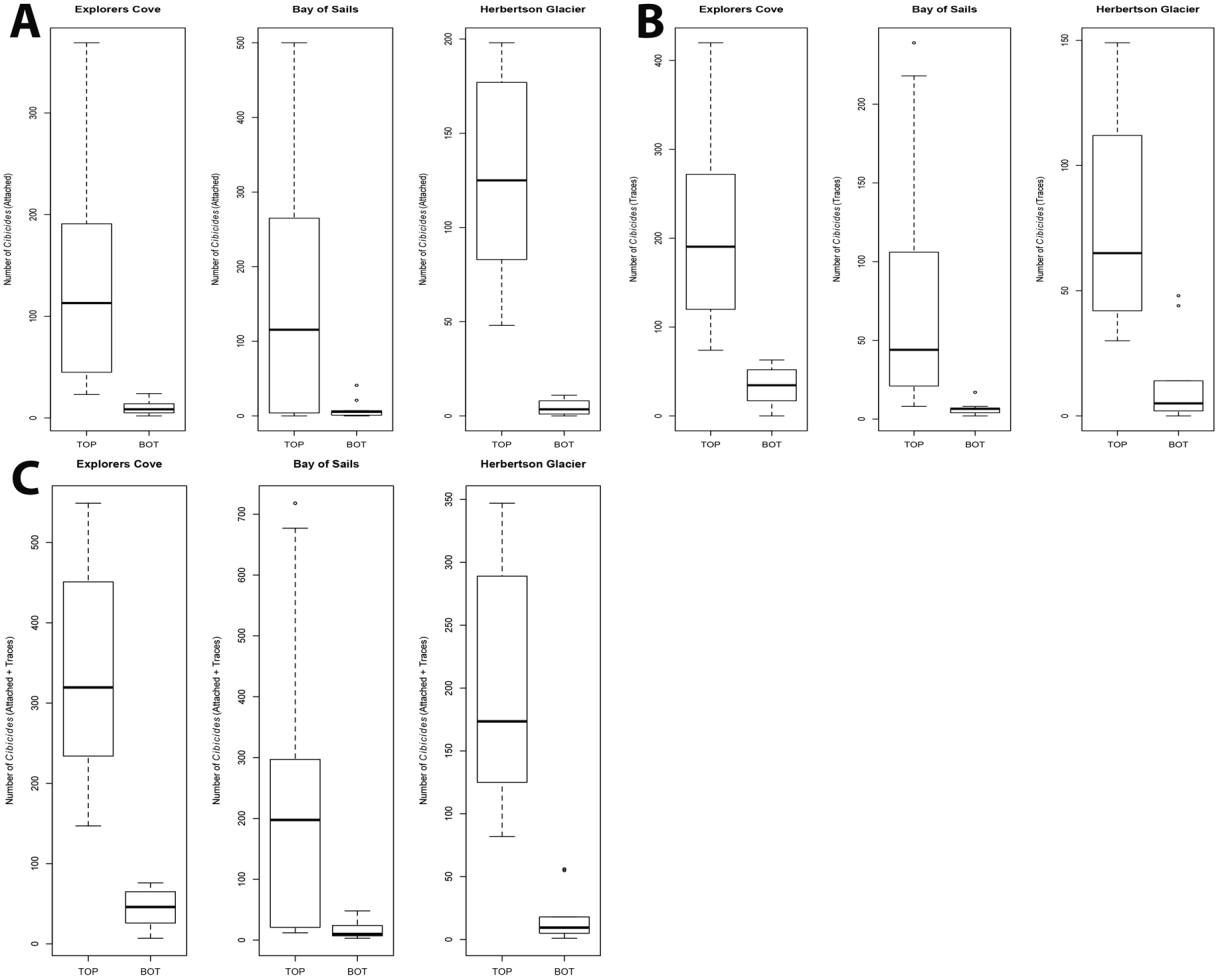
*Cibicides* Populations on Top and Bottom Valves by Locality. A. Attached *Cibicides*. B. *Cibicides* traces. C. Attached *Cibicides* with traces pooled. Top = top valves; Bot = bottom valves of *Adamussium colbecki*.

Top valves had the largest attached *Cibicides* populations compared to bottom valves for all three localities (Fig 5A. EC: *t* = 3.69, df = 9.08, *p* = 0.004; BOS: *t* = 2.767, df = 9.09, *p* = 0.02; HG: *t* = 6.905, df = 9.07, *p* < 0.0001). Similarly, traces were more common on top valves (Fig 5B; chi-square χ^2^ 24.956<1). Overall, top valves yielded 91% of living and trace *Cibicides* populations than bottom valves and this was significant for all localities (*Cibicides* + traces; Table 1, Fig 5C; EC: *t* = 7.117, df = 9.59, *p* < 0.0001; BOS: *t* = 2.825, df = 9.05, *p* = 0.02; HG: *t* = 6.141, df = 9.88, *p* = 0.0001).

**Fig 6:**
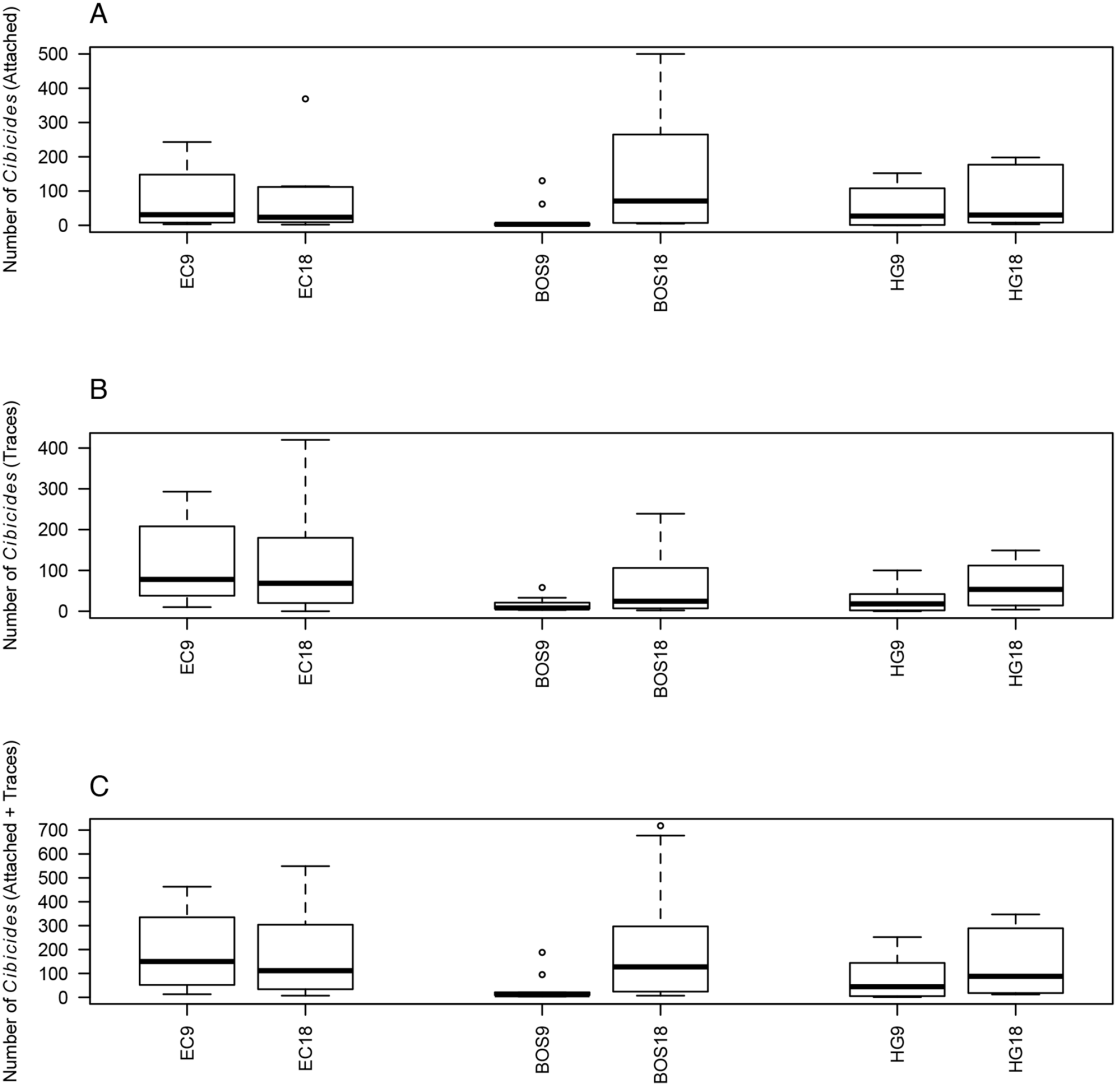
*Cibicides* Populations by Depth and Locality. A. Attached *Cibicides*. B. *Cibicides* traces. C. Pooled *Cibicides* with traces. Localities: Explorers Cove (EC), Bay of Sails (BOS), and Herbertson Glacier (HG). Depths are 9 and 18 m.

In general, attached *Cibicides* and their traces did not vary with depth. Attached *Cibicides* populations were not significantly different with depth, except at BOS that had a significantly higher population at 18 m than 9 m (Fig 6A; BOS *t* = -2.226, df = 37.99, *p* = 0.03). Traces were not significantly different with depth for all localities (Fig 6B). When *Cibicides* and traces were pooled, there was no difference with depth at EC and HG, except at BOS that had a significantly larger pooled population at 18 m (Fig 6C; BOS: *t* = -2.699, df = 37.74, *p* = 0.01).

### Parasite load

**Fig 7:**
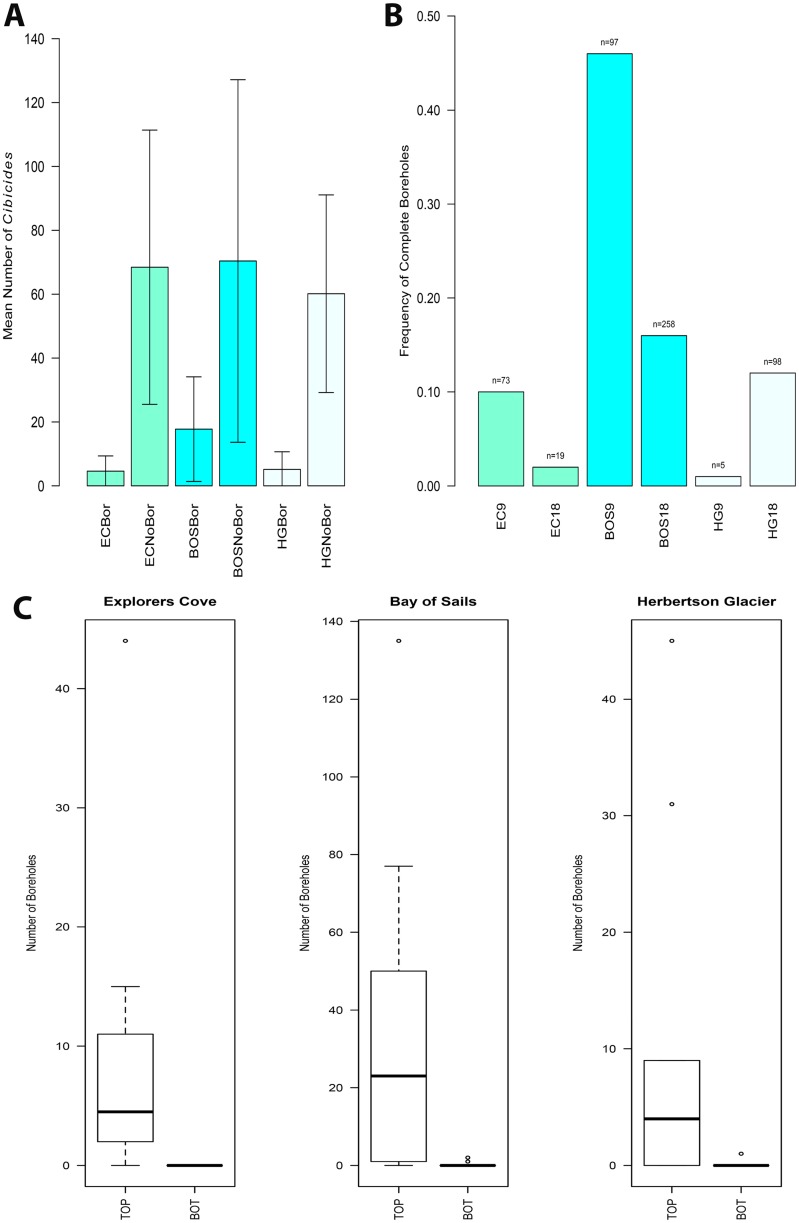
*Cibicides* Parasite Load by Locality, Depth and Valve Type. For A and B, localities are Explorers Cove (EC), Bay of Sails (BOS), and Herbertson Glacier (HG). A. Mean number of complete boreholes (Bor) compared to *Cibicides* without boreholes (NoBor), error bars are 95% CIs. B. Frequency of *Cibicides* boreholes by locality and depth. Frequency was calculated by dividing the number of *Cibicides* boreholes by the total population of *Cibicides*. C. Boreholes on top and bottom valves by locality.

Parasite load was relatively low for two of the three localities based on the mean number of *Cibicides* (Fig 7A). Parasitic *Cibicides* were significantly less common than non-parasitic *Cibicides* at EC and HG, but at BOS there was no difference between these groups. In general, the localities were not significantly different from each other for the mean number of parasitic and non-parasitic *Cibicides*.

The frequency of parasitic *Cibicides* varied by depth at each locality, ranging from 2 to 50% (Fig 7B). Parasitic *Cibicides* were significantly more common at 9 m for EC and BOS, but not at HG where significantly more parasites occurred at 18 m (FET: EC, *p* < 0.0001; BOS *p* < 0.00001; HG *p* < 0.00001). Nearly 50% of the *Cibicides* population at BOS 9 m was parasitic compared to 10% at EC and 2% at HG. The high number of boreholes at BOS 9 m, however, is biased by one shell that had 68 boreholes out of a total of 130 *Cibicides*. The 18 m depth at BOS and HG had more parasitic *Cibicides* (16% and 13%, respectively) than EC (10%).

Parasitic *Cibicides* were more common on top valves than bottom valves at all three localities (Fig 7C). This result was significant for all localities except HG because of the low number of boreholes at that locality (FET: EC, *p* = 0.002; BOS, *p* < 0.00001).

### *Cibicides* population structure and spatial distribution

#### Population structure

**Fig 8:**
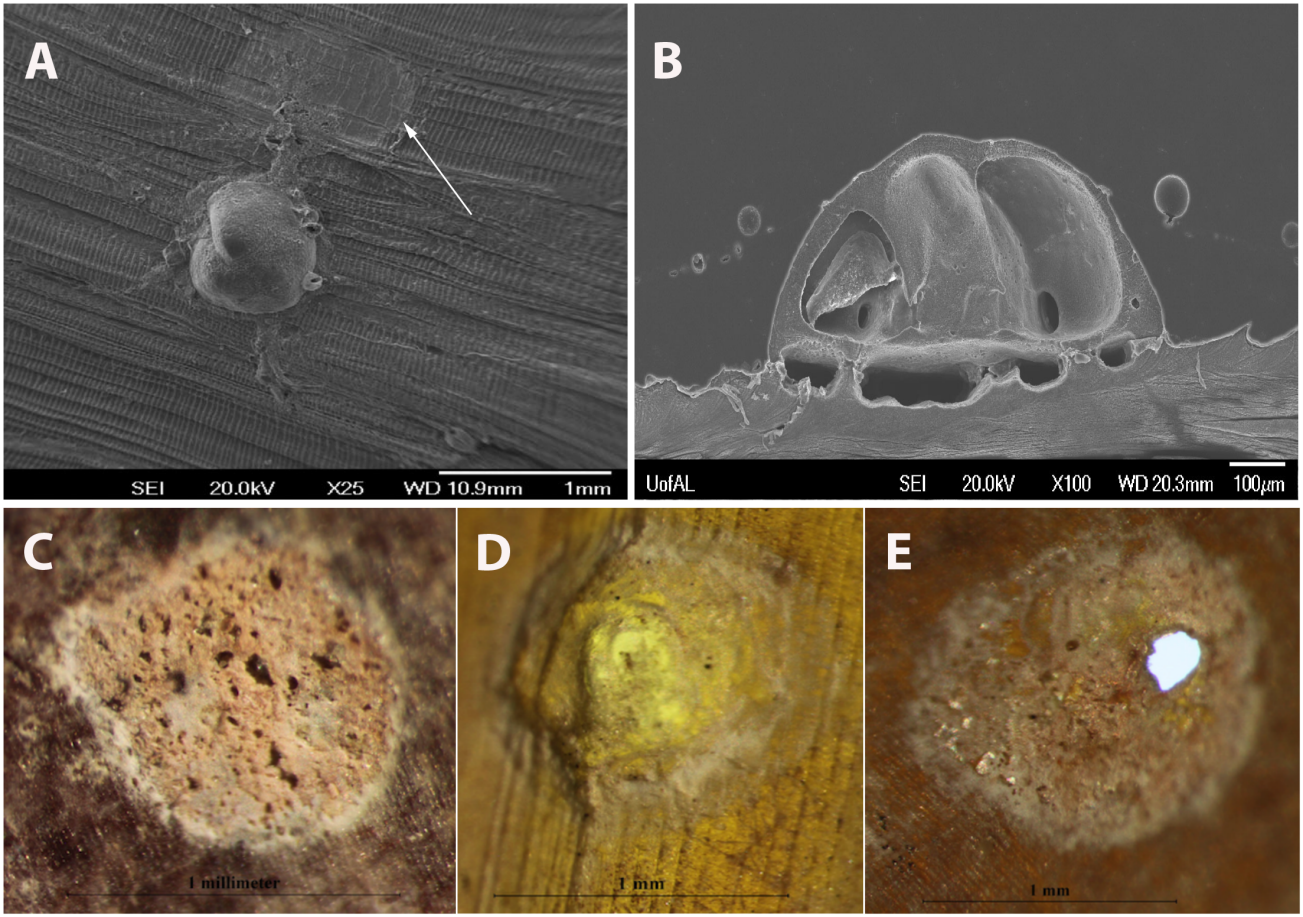
Four Bioerosion Trace Types Representing Ontogenetic Stages used to Examine *Cibicides* Population Structure. Scanning electron micrographs are depicted in A-B, and light photomicrographs are depicted in C-E. A. Attached *Cibicides* with agglutinated feeding tubes and an initial resting trace (trace type 1) etched by a *Cibicides* (arrow). B. Attached *Cibicides* cut in half to reveal the etched upper calcite layer of *Adamussium* representing trace type 2. C. Trace type 2 with multiple small holes. D. Trace type 3 with an incomplete borehole. E. Trace type 4 with a complete borehole representing parasitic *Cibicides*.

*Cibicides* bioerosion traces at EC and BOS were categorized into ontogenetic stages that revealed the age structure of the population. The traces ranged from small recruits that first etched the scallop’s shell to larger, parasitic adults that made complete boreholes. Four different ontogenetic stages of *Cibicides* traces were recognized. Trace 1 (T1) has a relatively smooth resting trace that represents juvenile *Cibicides’* early attachment to the shell (Fig 8A). Trace 2 (T1) has a resting trace punctuated by multiple small holes (Fig 8B and 8C). Trace 3 (T3) has a resting trace with one incomplete borehole (Fig 8D). Lastly, Trace 4 (T4) has a resting trace with a complete borehole (Fig 8E). Small accessory holes as depicted in Fig 8C can also be present in T3 and T4.

Young *Cibicides* were most common at both localities. The early trace types T1 and T2 represent 32% and 54% of the pooled EC + BOS traces, respectively (Table 2). The later ontogenetic stages of T3 and T4 were much less common representing 12% and 2%, respectively, of the pooled trace population. The mean spatial area of the traces increased from T1 (0.58 mm^2^) to T3 (1.23 mm^2^). Trace type T4 had a mean spatial area of 1.13 mm^2^.

**Table 2:** *Cibicides* Bioerosion Traces Categorized into Ontogenetic Trace Types. The trace types were categorized based on trace diameter and the degree of bioerosion. The trace type range from initial recruits that started to etch the shell (T1) to parasitic adults with complete boreholes (T4).

Trace Type	N	Frequency	Mean/SD	Mean Diameter (mm^2^)	SD Diameter (mm^2^)
T1	1529	0.32	23/21	0.58	0.37
T2	2568	0.54	39/45	0.82	0.33
T3	582	0.12	9/7	1.23	0.45
T4	78	0.02	1/2	1.13	0.37

N, abundance of trace types pooled from six *Adamussium* valves representing Explorers Cove and Bay of Sails localities; frequency, frequency of occurrence (out of total trace types pooled); SD, standard deviation; diameter refers to the outer bioerosion trace diameter.

**Fig 9:**
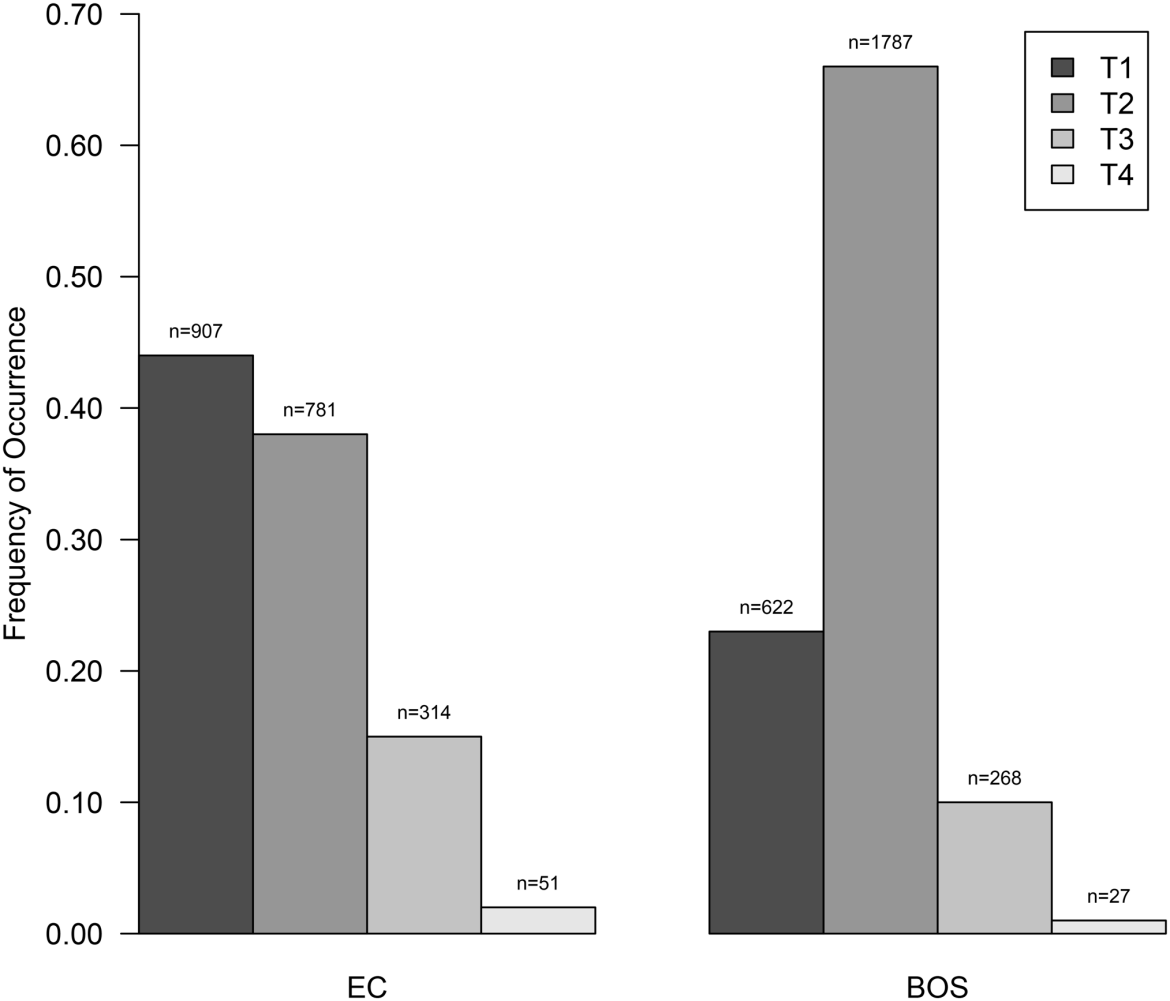
*Cibicides* Population Structure based on Trace Type for Explorers Cove and Bay of Sails. Trace type T1-T4 as depicted in Fig 8. EC, Explorers Cove; BOS, Bay of Sails. The sample size for each trace is provided above each bar in this bar plot.

Population structure varied within and between localities (Fig 9). At EC, new recruits (T1) represented 44% of the trace population followed by T2 with 38%, T3 with 15% and T4 with 2%. BOS had fewer new recruits (T1) representing only 23% of the trace population. At BOS, the next ontogenetic stage (T2) represented 66% of the trace population followed by T3 with 10% and T4 with 1%. Most of these trace types were significantly different from each other (S1 Table). The age structure of the population was significantly different between EC and BOS (K-W test: chi-square = 128.42, df = 3, *p* < 0.0001). This difference results from BOS having significantly more T2 traces than EC (W = 319, *p* = 0.003, at alpha = 0.0125).

#### Spatial distribution

**Fig 10:**
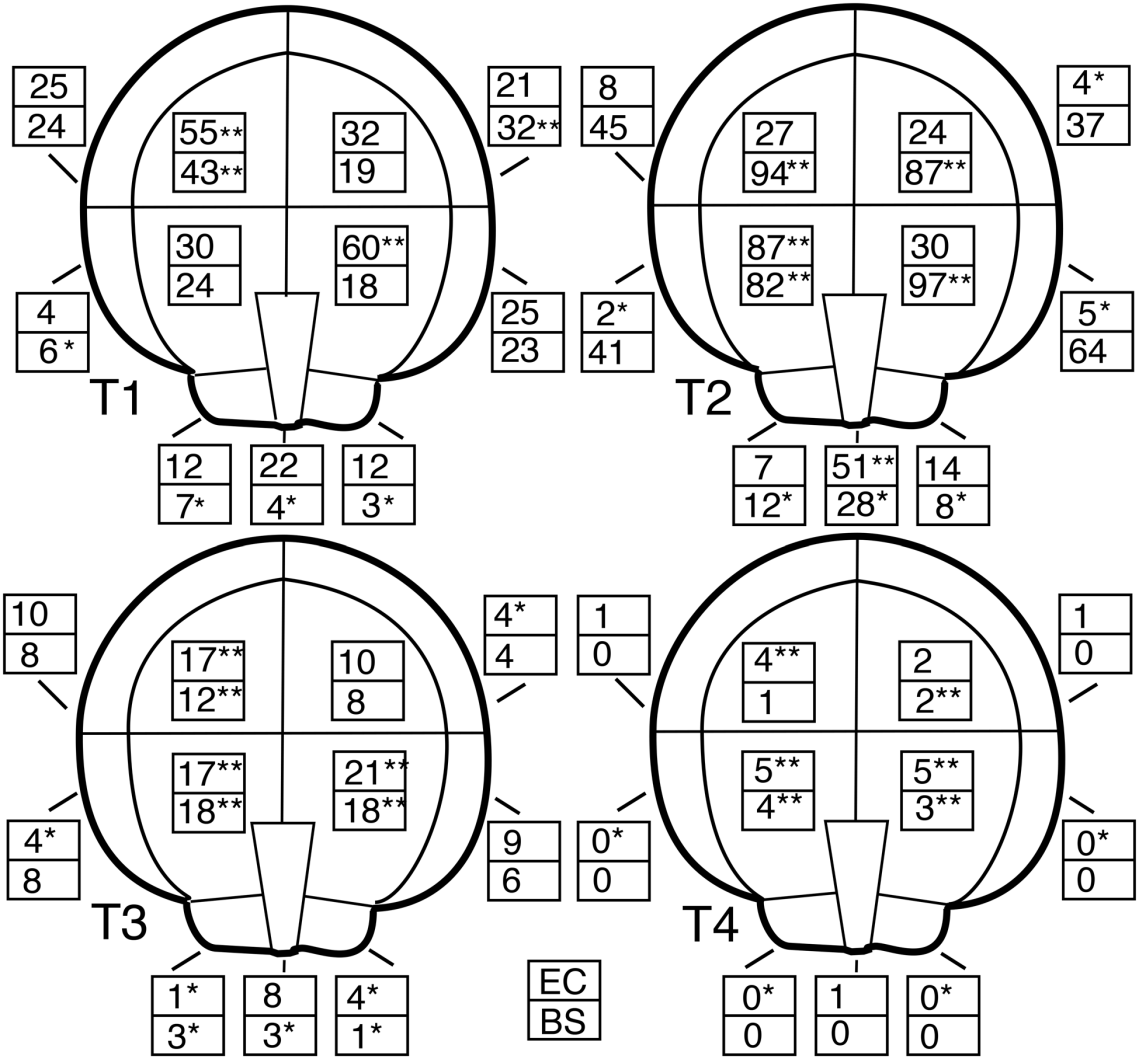
Trace Type by Shell Sector for Explorers Cove and Bay of Sails. The mean number of trace types per shell sector are depicted. The mean was adjusted for sector area. EC, Explorers Cove; BS, Bay of Sails. ** indicates a significantly larger mean population occurs in the sector; * indicates a significantly lower mean population occurs in the sector. Significance is based on 95% CIs generated by one-sample *t*-tests used for each trace type.

The categorized traces were also used to examine the spatial distribution of *Cibicides* ontogenetic stages on the top valves of *Adamussium*. Trace types at EC and BOS were more common in the central region of the scallop than the edges (Fig 10). In general, all trace types had significantly higher mean densities in the center sectors of the shell than the outer sectors (Fig 10; S2 Table). Traces with incomplete boreholes (T3) and complete boreholes (T4) were especially common in the center sectors overlying the muscle, gonads, and other soft tissue (sectors 4, 5). A few of the trace types occurred in some outer sectors and the umbo, but they were not as common as those in the center region of the shell.

### CaCO_3_ biomass and annual production

**Fig 11:**
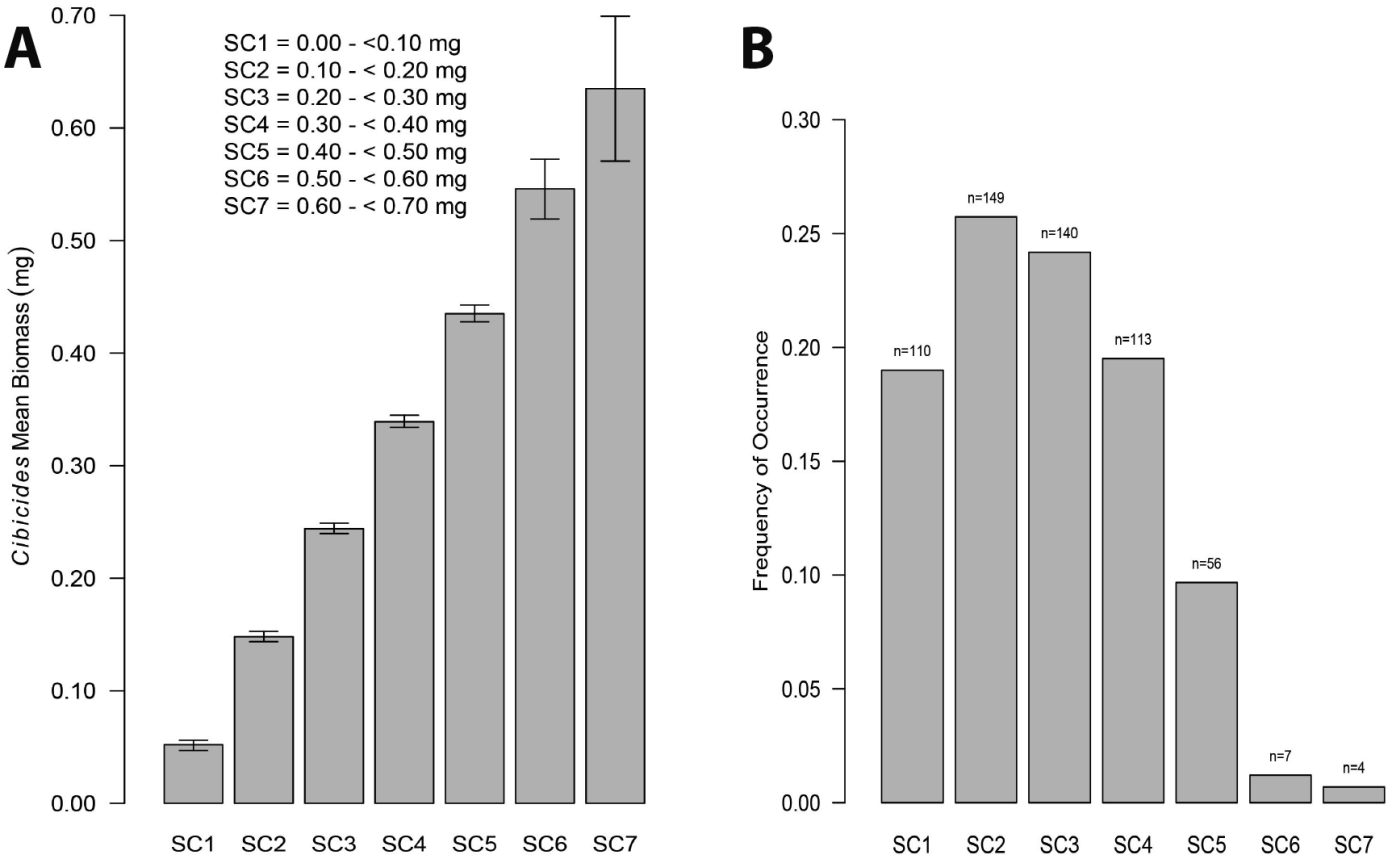
Size Class and Frequency for *Cibicides* CaCO_3_ Biomass. A. Mean CaCO_3_ biomass by size class with 95% CIs. Biomass size classes (SC) ranged between 0.00–0.70 mg. B. CaCO_3_ biomass size class frequency. The sample size for each size class is reported above the bars in the bar plot.

Mean CaCO_3_ biomass for attached *Cibicides* ranged from < 0.001 mg to 0.690 mg and represented seven size classes (Fig 11A). All biomass classes were significantly different from each other except for the two largest classes (SC 6–7). The mean CaCO_3_ biomass was 0.226 mg and was used to calculate CaCO_3_ biomass density and annual CaCO_3_ production. The frequency distribution for CaCO_3_ biomass was positively skewed toward younger individuals (Fig 11B).

**Fig 12:**
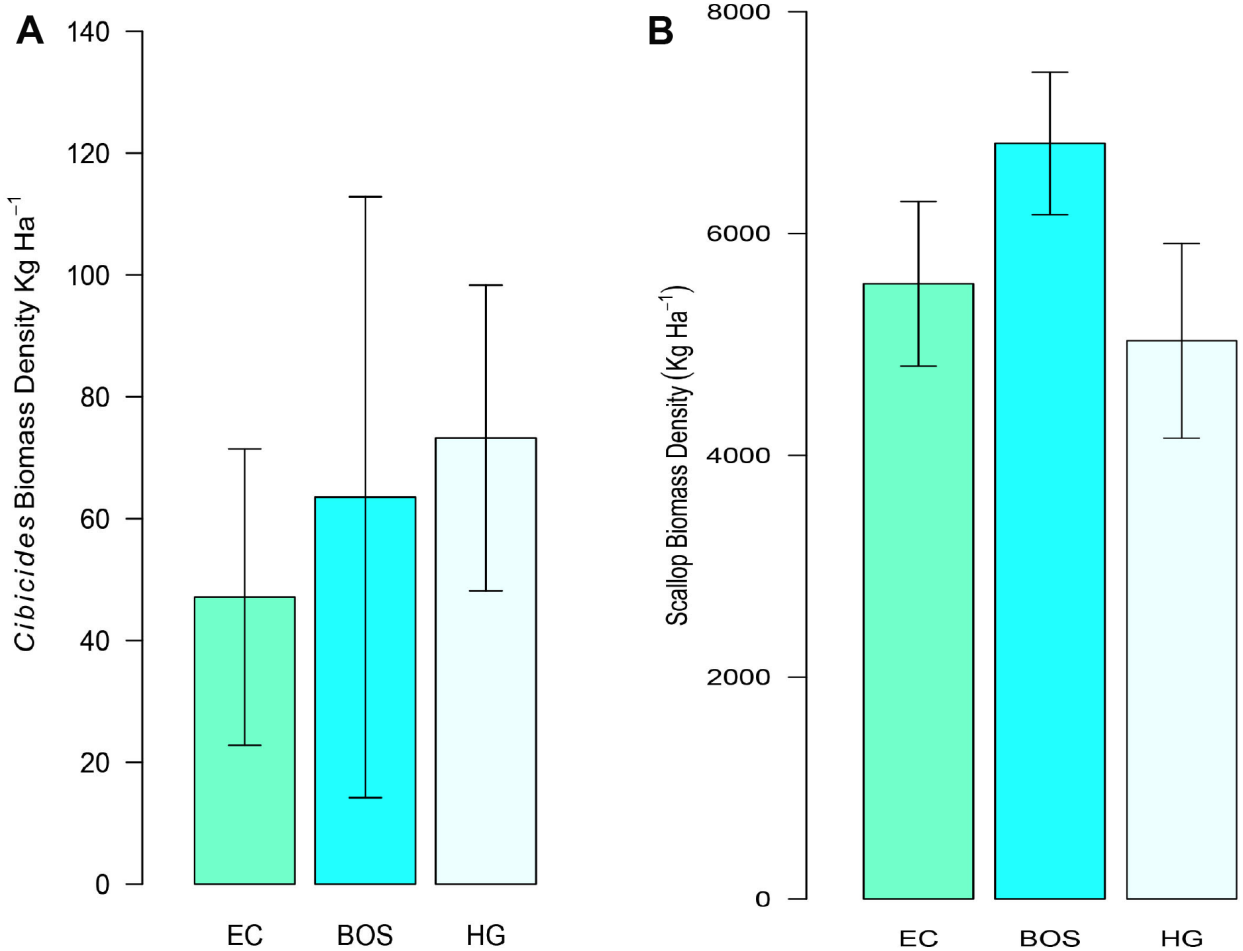
CaCO_3_ Biomass Density for *Cibicides* and *Adamussium* by Locality. The mean CaCO_3_ biomass density is depicted with 95% CIs. Localities are Explorers Cove (EC), Bay of Sails (BOS) and Herbertson Glacier (HG). A. CaCO_3_ biomass density for attached *Cibicides*. B. CaCO_3_ biomass density for *Adamussium*.

Localities were similar in mean biomass density for both attached *Cibicides* and *Adamussium*. Because HG had the highest mean density of *Cibicides*, it also had the largest CaCO_3_ biomass density, 73 kg ha^-1^, followed by BOS with 64 kg ha^-1^ and EC with 47 kg ha^-1^, though the differences were not significant (Fig 12A). *Adamussium* CaCO_3_ biomass density was > 5000 kg ha^-1^ for all localities, and the localities were not significantly different from each other (Fig 12B).

**Fig 13:**
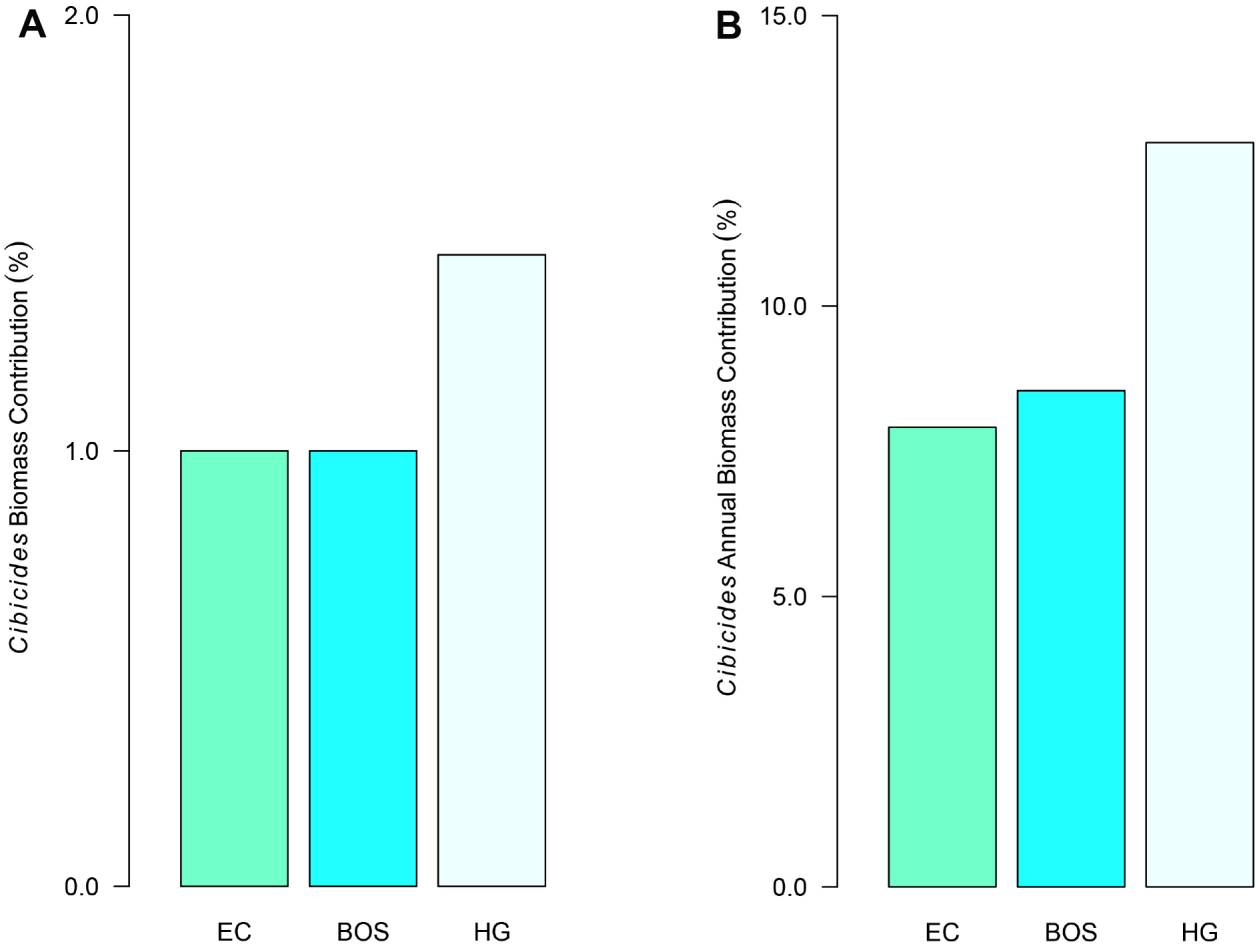
*Cibicides* Contribution to Annual CaCO_3_ Production by Locality. *Cibicides* percentage is based on the amount of CaCO_3_ it produces out of the total *Cibicides* and *Adamussium* biomass at each locality. Localities are Explorers Cove (EC), Bay of Sails (BOS) and Herbertson Glacier (HG). A. CaCO_3_ biomass contributed by attached *Cibicides*. B. Yearly production of CaCO_3_ by attached *Cibicides*.

*Adamussium* contributed three orders of magnitude more CaCO_3_ to our Antarctic localities than *Cibicides*. However, the CaCO_3_ biomass contributed by *Cibicides* was not trivial. *Cibicides* biomass represented 1% of the total CaCO_3_ produced by both species at EC and BOS and 1.5% at HG (Fig 13A). *Cibicides* yearly CaCO_3_ production ranged from 8 to 13% of that generated by the species pair depending on locality (Fig 13B). Annual CaCO_3_ production by *Cibicides* varied among the localities from 24 kg ha^-1^ yr^-1^ at EC to 37 kg ha^-1^ yr^-1^ at HG (Table 3). *Adamussium* CaCO_3_ biomass also varied across localities, ranging from 249 kg ha^-1^ yr^-1^ at HG to 340 kg ha^-1^ yr^-1^ at BOS (Table 3). Together, *Adamussium* and attached *Cibicides* contribute from 286 to 372 kg ha^-1^ yr^-1^ of CaCO_3_ to our Antarctic localities (Table 3).

**Table 3:** Annual CaCO_3_ Production for *Cibicides antarcticus* and *Adamussium colbecki* by Locality. *Cibicides* estimates are based on the conservative mean CaCO_3_ biomass of 0.226 mg.

Taxon	Explorers Cove (kg ha^-1^ yr^-1^)	Bay of Sails (kg ha^-1^ yr^-1^)	Herbertson Glacier (kg ha^-1^ yr^-1^)
*Cibicides*	24	32	37
*Adamussium colbecki*	274	340	249
Total mean *Cibicides* and *Adamussium*	298	372	286

## Discussion

### Living *Cibicides*

It is essential to distinguish between living and dead populations of *Cibicides* for population analysis and carbonate estimates. An *in situ* experiment by SSB showed that attached *Cibicides* represent living individuals. This finding is in agreement with preliminary observations that > 98% of attached *Cibicides* on other *Adamussium* shells were living based on staining with CellTracker Green (SSB personal observation). Therefore, attached *Cibicides* represent living individuals and their traces represent former populations of *Cibicides* that are no longer attached to the shell.

### *Cibicides* populations by locality, valve type, and depth

*Cibicides* populations varied by locality, valve type, and depth. BOS had the largest population of attached *Cibicides* followed by EC and HG. Densities of attached *Cibicides* were highest at the two annual sea ice localities BOS and HG. By comparison, the multiannual sea ice locality (EC) had the lowest density of attached *Cibicides*. Yet, when attached *Cibicides* were pooled with traces, EC had the largest total population, followed by BOS and HG.

Population differences among the localities could be related to the host’s age: the older the scallop, the longer *Cibicides* can accumulate on the shell, thereby increasing population size. The size of *Adamussium* is used as an age equivalent [55, 57]. If we compared our shell sizes to a von Bertalanffy growth equation generated by [56], the approximate age for EC and BOS scallops would be ~15 years and ~12 years for HG. If these age estimates are correct, the total *Cibicides* population (attached *Cibicides* + traces) should be similar between EC and BOS with a smaller population at HG. That was not the case: EC had significantly higher total populations than BOS. The results from the GLM also revealed that attached *Cibicides* populations did not increase with shell area despite HG and BOS having slightly smaller and larger shells, respectively. Other factors must be contributing to *Cibicides* population dynamics.

Alternatively, a higher total population of *Cibicides* at EC could be related to sea ice cover. EC was the only locality with persistent sea ice cover, lasting at least a decade prior to our study, whereas sea ice melts out every year at BOS and HG. Sea ice algae are strongly tied to sea ice cover, often reaching concentrations up to hundreds of mg m^-3^ [30]. Sea ice and its associated microbial communities provide nutrients for Antarctic benthic organisms during austral summer and winter [30–31]. As a result, Antarctic benthic communities are strongly affected by sea ice algae distribution, as well as water column and benthic primary productivity [38, 46]. In a study on trophic associations, *A*. *colbecki* and the echinoid *Sterechinus neumayeri* had delta ^15^N signatures indicative of a diet rich in sea ice algae and benthic detritus at EC. At Terra Nova Bay, where sea ice melts out every year, *Adamussium* fed on phytoplankton and *Sterechinus* fed on benthic seaweeds [58]. Additionally, studies in the Bering Sea have shown that a loss of sea ice leads to a decrease in benthic biomass and a shift towards pelagic ecosystems [59]. These results suggest that fluctuations in annual sea ice are related to the strength of trophic interactions. Larger total *Cibicides* population at EC could be linked to increased nutrients from sea ice algae, enhancing suspension feeding and grazing in this species.

Top valves had the largest *Cibicides* populations compared to bottom valves. Bottom valves have smaller populations because part of the valve rests on the seafloor, limiting *Cibicides* settlement. Frequent valve clapping by *Adamussium* likely increases abrasion on bottom valves reducing foraminifera populations [28, 60]. Moreover, top valves are elevated above the sediment-water interface. *Cibicides* species tend to live on elevated surfaces where increased water flow enhances suspension feeding [61]. Valve clapping also stirs up fine seafloor sediment that could facilitate suspension feeding in *C*. *antarticus* that live on top valves [51].

*Cibicides* populations were similar at 9 m and 18 m for all localities except BOS, which had significantly higher attached *Cibicides* populations at 18 m. This difference could result from the patchy nature of foraminiferal distributions on *Adamussium* valves [26]. It could also result from shallow water disturbance, possibly from anchor ice. Anchor ice occurs to water depths of ~ 33 m in Antarctica, but recent research suggests that anchor ice can occur at much deeper depths [62–63]. Anchor ice grows on sediment and benthic organisms [62]. If *Adamussium* valves were affected by anchor ice at BOS 9 m, we suggest that this could reduce *Cibicides* populations. Differences in recruitment patterns, predation, or other factors could also account for the reduced populations at BOS 9 m.

Mean densities of *C*. *antarcticus* at our Antarctic localities are higher than for other *Cibicides* species living in Arctic and tropical regions [52, 64]. For example, mean densities of *Cibicidoides* (*Cibicides*) *lobatulus* ranged from 0.33 cm^-2^ to 2.32 cm^-2^ on experimental plates deployed off Kosterfjord, southwestern Sweden (converted from m^2^, Appendix 1 in [52]). In the tropical Caribbean, *Cibicides* species that encrusted experimental shells after two years on the sea floor had mean densities of ~ 4 cm^-2^ (15 m depth in [64]). These lower mean densities may be related to increased competition with other encrusting species [65], predation [12], or other factors. For *C*. *antarcticus*, its multiple trophic modes could allow it to grow larger and therefore out compete other shell-encrusting foraminifera or invertebrates for resources and space. This species has agglutinated feeding tubes that extend horizontally from its test [13], which could also increase its effectiveness in competing for space.

### Parasite load

Parasite populations vary widely across spatial scales in a single host [66]. These differences could be caused by biotic and abiotic environmental conditions [67]. Parasite load in *Cibicides* also varies widely across our Antarctic localities despite sharing the same host. The overall parasite load varied between 2% and 50% depending on locality and depth at our Antarctic localities. The original work on *Cibicides* parasitism found that ~ 50% were parasitic at EC [13]. We found that for most localities, parasitism was < 20%.

Abiotic environmental conditions could have changed since the original work on parasitism in Antarctic *Cibicides*. That study was conducted during a time of more frequent sea ice melt outs that possibly affected foraminiferal populations [26]. If sea ice loss is a factor, then *Cibicides* may resort to increased parasitism as a trophic mode to supplement its nutrition. At BOS, one of the annual sea ice localities, the parasite load was much higher than EC. Yet at the other sea ice locality HG, the parasite load was similar to EC at 18 m but much lower at 9 m. Rather, it appears that the parasite load is likely related to the population structure of *Cibicides* and its patchy distribution than sea ice conditions. Adult *Cibicides* are parasitic and the parasite load is most likely a function of the number of adults in the population.

Differences in methods between the original study on *Cibicides* parasitism and ours could also account for variation in parasite load. In the original study, they counted the number of etched traces as an indication of parasite load [13], while we counted complete boreholes. Because the number of etched traces are far more common that complete boreholes, they could be overestimating parasitism. They did demonstrate that one etched trace had multiple minute borings that penetrated almost to the shell interior [13]. We could have missed these small pseudopodial borings that do not elicit a host response. By this measure we could be underestimating the parasite load at our localities, suggesting that our estimates are conservative.

Bioerosion is considered a form of parasitism when it contributes to weakening mollusc shells [68]. Boreholes of *Cibicides* and associated bioerosion traces could negatively affect *Adamussium*. Considering that *Adamussium*’s shell is ~ 0.70 mm thick, *Cibicides* etchings and boreholes could weaken the shell, making it more susceptible to predators. Increased shell permeability also enhances dissolution. Additionally, there is a physiological cost for the scallop because it forms blisters in response to *C*. *antarcticus* boreholes, although not all complete boreholes were associated with blisters (SEW personal observation). Shell weakening, increased dissolution and the physiological expense of shell repair could all be added costs of high *Cibicides* populations on *Adamussium*.

Parasitism could have arisen accidently in *C*. *antarcticus* as a result of the thin shell of *Adamussium*. Given enough time to bioerode the shell, *Cibicides* could penetrate to the shell interior. For example, closely related cibicidids living on thicker carbonate substrates, like *C*. *refulgens* [69] and the more distantly related *C*. *lobatulus*, bioerode but are not parasites [52, 64]. If *C*. *antarcticus* lives for two years or longer, its bioerosive activities could completely penetrate the thin shell of *Adamussium*. The uptake of nutrients and minerals would be beneficial to *Cibicides* in Antarctic environments where conditions are similar to the deep sea. Further studies are needed to address *Cibicides* bioerosion rates and whether they use CaCO_3_ from *Adamussium*, as well as determine whether *Adamussium* populations are affected by large populations of *Cibicides*.

### *Cibicides* population structure and spatial distribution

#### Population structure

Bioerosion traces made by *C*. *antarcticus* not only provide a record of the parasite load, but also reveal information about its population structure, recruitment patterns, and spatial distributions. Four *Cibicides* bioerosion traces were documented on *Adamussium* shells from EC and BOS. These traces represent ontogenetic stages from newly attached individuals that had just started to etch the shell (T1) to parasitic adults with complete boreholes (T4). Overall, early stage traces (T1-T2) were more common than later stage traces (T3-T4) at both localities, indicating that *Cibicides* populations represent mostly younger individuals. We concur with Alexander and DeLaca’s [13] conclusions that adult *C*. *antarcticus* are parasitic, because incomplete and complete boreholes only occurred with larger individuals.

Traces revealed differences in recruitment patterns at the two Antarctic localities. EC had mostly newly attached individuals (T1), while BOS had significantly more of the next stage trace (T2) that had small holes etched into the shell surface. EC also had more adults (T3 and T4) than BOS, but the difference was not significant. The variation in trace population structure suggests differences in *Cibicides* reproduction and recruitment between EC and BOS. Perhaps sea ice algae and their associated nutrients facilitate higher reproductive rates for *Cibicides* at EC. Fluxes in sea ice nutrients could also contribute to higher population turnover at EC, which could account for the higher number of traces at this locality. *Cibicides* also encrusts seafloor glacial erratics at EC and also occurs in sediments although these are likely dead individuals [SSB personal observation]. The population structure of *Cibicides* on glacial erratics needs to be compared to those on *Adamussium* valves. If population structure and test size are similar among these different substrates, then parasitism may not be that important for *Cibicides*.

#### Spatial distribution

Parasites have heterogeneous distributions in host populations that can be spatially structured [70]. Spatial distributions of *Cibicides* on *Adamussium* valves could reveal preferred settlement sites that maximize food capture. If suspension feeding is important, *Cibicides* ought to attach where feeding currents are higher presumably near the scallop’s aperture. For example, fossil foraminifera preferentially encrust brachiopod apertures, presumably to take advantage of their feeding currents [71]. However, *C*. *antarcticus* is thought to have random distributions on *Adamussium* valves [51], suggesting that water currents generated by the scallop are not important. We found that *Cibicides* traces were significantly more common in the center region of *Adamussium*’s top valves. We also found that center sectors had more parasitic boreholes than the outer sectors. Rarely were complete boreholes found outside of these sectors. More trace populations are likely in the center sectors because they represent some of the oldest regions of the shell, allowing more time for several *Cibicides* generations to accumulate. Furthermore, these sectors are located over the muscle, gill and gonadal tissue, and perhaps parasitic *Cibicides* are targeting those tissues.

### CaCO_3_ biomass and annual production

We estimated the mean CaCO_3_ biomass for *C*. *antarcticus* and its host *Adamussium* for our localities. We first showed that *Cibicides* had a CaCO_3_ biomass that ranged from < 0.001 to 0.690 mg, with a mean of 0.226 mg. This mean is an order of magnitude higher than that reported for non-parasitic *Cibicidoides lobatulus* from polar waters near Kosterfjord, southwestern Sweden (0.0187 mg in [52]). Unfortunately, CaCO_3_ biomass is not reported for other parasitic foraminifera, so we cannot compare our estimates. At our Antarctic localities, *Cibicides* CaCO_3_ biomass varied from 47–73 kg ha^-1^.

We next demonstrated that *Adamussium* shells were almost pure carbonate and therefore can be used as a CaCO_3_ biomass proxy. Mean CaCO_3_ biomass from *Adamussium* varied from 4987–6806 kg ha^-1^ at our localities. The mean CaCO_3_ biomass per hectare for both *Cibicides* and *Adamussium* was not significantly different across our localities.

*Cibicides* contributes a non-trivial amount of CaCO_3_ to the host-parasite relationship and represents contributions from younger individuals. *Cibicides* mean CaCO_3_ biomass represents 1.0–2.3% of the total CaCO_3_ biomass produced by *Cibicides* and *Adamussium* combined. These estimates are similar to biomass contributions for soft-bodied parasites of metazoans (crabs, polychaetes, bivalves, birds) from subtropical to warm-temperate estuaries [4]. Additionally, *Cibicides* CaCO_3_ biomass at EC and BOS represents mostly young individuals. A survivorship curve using biomass revealed that *Cibicides* has a Type I curve, indicative of low juvenile mortality and increasing mortality with age. Most benthic foraminifera have a Type III curve characterized by high juvenile mortality with few surviving to adulthood [72].

Annual CaCO_3_ production rates for *C*. *antarcticus* are relatively high compared to *C*. *lobatulus* from near-Arctic polar regions. Antarctic *Cibicides* contributes from 24–37 kg ha^-1^ yr^-1^ of CaCO_3_ to our three localities. In near-Arctic polar regions, non-parasitic *C*. *lobatulus* contributes 0.00–0.32 g m^-2^ yr^-1^ of CaCO_3_ (3.26 kg ha^-1^ yr^-1^ in [52]). This near-Arctic species produces an order of magnitude less than Antarctic *Cibicides* even though the former has much larger populations.

*Cibicides* and *Adamussium* likely contribute considerable CaCO_3_ to Antarctic communities where they occur. The annual CaCO_3_ production for *Adamussium* ranges from 249–340 kg ha^-1^ yr^-1^ and with *Cibicides*, adds 286–372 kg ha^-1^ yr^-1^ of CaCO_3_ to our Antarctic localities. Our estimates are conservative considering that *C*. *antarcticus* also lives on other hard substrates and its actual CaCO_3_ production could be far greater. Both these species are circum-Antarctic in distribution, although we know little about their populations except for a few localities in western McMurdo Sound. Based on our localities, they could potentially add 5.94 x 10^9^ kg ha^-1^ yr^-1^ of CaCO_3_ to the Ross Sea (assuming an areal extent of 1.87 x 10^7^ ha). Future studies need to address their CaCO_3_ biomass at other localities to bracket their contributions to the carbonate budget of the Ross Sea.

Even though the CaCO_3_ biomass contribution of *Cibicides* and *Adamussium* is considerable, the Ross Sea has very little carbonate in the sedimentary record, a direct result of high primary productivity [73]. Although increased primary productivity contributes to high diversity in this region, carbonate skeletons are rarely preserved in the sediment. High acidic porewaters resulting from benthic respiration induced by primary productivity is inimical to carbonate preservation [37, 73]. Thus, carbonate production by *Adamussium*, *Cibicides*, and other carbonate organisms are recycled quickly back into the Ross Sea once they are buried, which could, in part, maintain the supersaturated levels of CaCO_3_ in this region. This should not preclude work on carbonate producers, as we need to estimate the amount of carbonate produced by the high diversity of Ross Sea organisms to improve carbonate budgets in this region.

## Concluding Remarks

The facultative parasite *Cibicides antarcticus* and its Antarctic scallop host *Adamussium colbecki* are major components of Antarctic ecosystems, yet we know little about their populations. We found that *Cibicides* had large populations on *Adamussium* that varied by locality but not generally with water depth. The largest total *Cibicides* population, represented by attached individuals and bioerosion traces, occurred at EC. The EC locality has multiannual sea ice, distinguishing it from BOS and HG where sea ice melts out every year. Periodic pulses of sea ice algae could sustain the large populations at EC. *Cibicides* is a parasite but it also supplements its diet by suspension feeding and grazing on diatoms [13]. Suspension feeding might be more common in younger individuals, because adults are more commonly parasitic. However, even parasitic *Cibicides* suspension feed and graze on diatoms [13]. Future work using stable nitrogen isotopes could shed light on *Cibicides* trophic relationships.

Parasites are known to have heterogeneous populations in time and space and *Cibicides* does not deviate from this pattern. *Cibicides* parasite load varied by locality, ranging from 2% to 50% of *Cibicides* populations. Overall, parasitism was rare, occurring in <20% of the population for all localities except for the 9 m site at BOS with the highest parasite load of 50%. Previously, parasitism was thought to occur in ~50% of *Cibicides* populations at EC [13]. These differences could be related to temporal changes in the environment since that study was conducted. At the time of the original study in the 1980s, sea ice melt out was more common at EC [26]. Since then, sea ice has persisted with occasional partial melt outs. Parasitism in *Cibicides* could be less common in regions like EC that have persistent sea ice cover and associated sea ice algae. If sea ice begins to completely melt out at EC, it would be important to know if parasitism rates increase to the levels reported in the 1980s.

*Cibicides antarcticus* etches resting traces on the shell surface of *Adamussium* [13]. As *Cibicides* grows, it enlarges the trace and penetrates to the interior tissues of the scallop. As a result, the ontogenetic stages of *Cibicides* are recorded in these bioerosion traces as well as the development of parasitism. We categorized these ontogenetic stages and used them as a proxy for *Cibicides* population structure. Based on these traces, populations at EC represent recently attached *Cibicides*, while those on BOS shells represent slightly older populations. Large *Cibicides*, presumably adults, always had complete or incomplete boreholes. Therefore, differences in the parasite load among the localities could also be a function of a population structure skewed toward adult *Cibicides*. We propose that these trace categories can be used to examine the evolutionary history of the host-parasite relationship in recent death or fossil assemblages of *Adamussium* when *Cibicides* is no longer attached to the shell.

*Cibicides antarcticus* and *Adamussium colbecki* are major components of Antarctic ecosystems. We demonstrated that these species both contribute considerable amounts of CaCO_3_ to our Antarctic localities, potentially adding 5.94 x 10^9^ kg ha^-1^ yr^-1^ of CaCO_3_ to the Ross Sea. Because of their large CaCO_3_ contributions, they should be considered in southern polar food webs and ecosystem modeling. If we wish to better understand the global carbon and CaCO_3_ cycle, we also need to incorporate these species in Antarctic carbonate budgets.

## Supporting Information

S1 Fig Survivorship Curve for *Cibicides antarcticus*. The Type I survivorship curve is based on young to adult *Cibicides* biomass size classes. A Type I curve is characterized by high juvenile survivorship with increasing mortality with age.
(TIF)

S1 Table Within-Locality Significance Testing for Trace Types T1-T4. After an initial Kruskal-Wallis test, post hoc Wilcoxon rank sum tests with continuity correction were run to determine within-locality differences in trace type occurrence. Data were subset yielding an alpha = 0.008.
(DOCX)

S2 Table Trace Type 95% Confidence Intervals (CI). The CIs were used to determine if the mean number of *Cibicides* were significantly more or less common by shell sector. CIs were calculated using one-sample *t-*tests and the *t*-statistic is reported for each test. Trace type refers to *Cibicides* ontogenetic stages represented by their bioerosion traces. Trace types range from T1 (initial recruits that etch the shell surface) to T4 (parasitic adults that made complete boreholes in *Adamussium* valves).
(DOCX)
