# Supplementary figures and images for: Population Dynamics and Parasite Load of a Foraminifer on Its Antarctic Scallop Host with Their Carbonate Biomass Contributions
Source: PLoS One. 2015 Jul 17;10(7):e0132534. doi: 10.1371/journal.pone.0132534 (PMC4505869; doi:10.1371/journal.pone.0132534)

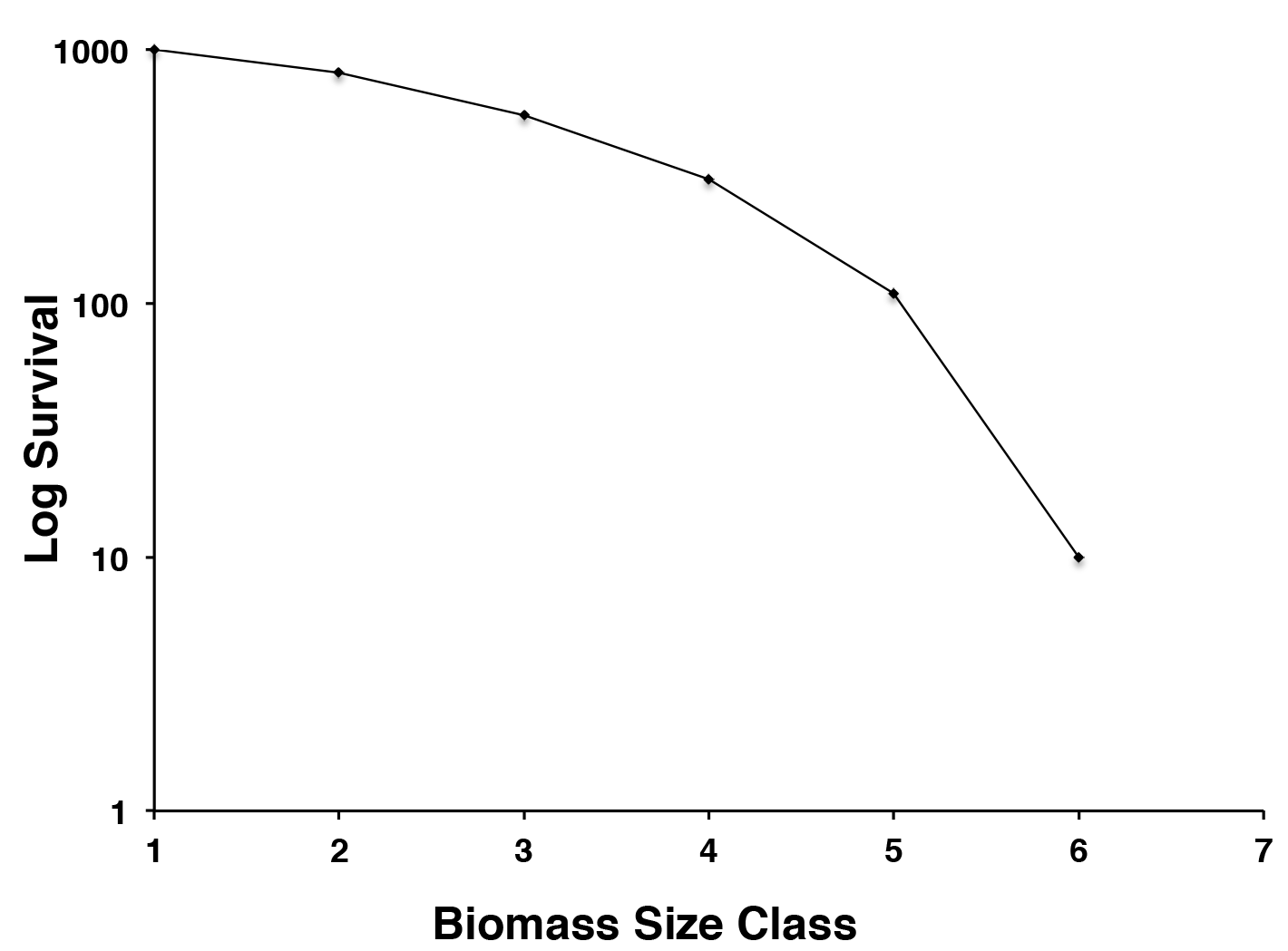

Supplement: S1 Fig — The Type I survivorship curve is based on young to adult Cibicides biomass size classes. A Type I curve is characterized by high juvenile survivorship with increasing mortality with age. (TIF) [file pone.0132534.s001.tif]
