# Supplementary material for: Population Dynamics and Parasite Load of a Foraminifer on Its Antarctic Scallop Host with Their Carbonate Biomass Contributions
Source: PLoS One. 2015 Jul 17;10(7):e0132534. doi: 10.1371/journal.pone.0132534 (PMC4505869; doi:10.1371/journal.pone.0132534)
Supplement: S1 Table — After an initial Kruskal-Wallis test, post hoc Wilcoxon rank sum tests with continuity correction were run to determine within-locality differences in trace type occurrence. Data were subset yielding an alpha = 0.008. (DOCX) [file pone.0132534.s002.docx]

| **Trace Type** | **T1** | **T2** | **T3** | **T4** |
| --- | --- | --- | --- | --- |
| Explorers Cove |  |  |  |  |
| T1 | -- | W = 299  *p* = 0.001 | n.s. | W = 1001  *p* < 0.0001 |
| T2 | -- | -- | W = 898.5  *p* < 0.0001 | W = 1068.5  *p* < 0.0001 |
| T3 | -- | -- | -- | W = 995.5  *p* < 0.0001 |
| Bay of Sails |  |  |  |  |
| T1 | -- | n.s. | W = 888.5  *p* < 0.0001 | W = 1064  *p* < 0.00001 |
| T2 | -- | -- | n.s. | W = 991.5  *p* < 0.00001 |
| T3 | -- | -- | -- | W = 965  *p* < 0.00001 |

W, post hoc Wilcoxon rank sum test statistic; a non-significant value is depicted as “n.s.”
