# Supplementary material for: Population Dynamics and Parasite Load of a Foraminifer on Its Antarctic Scallop Host with Their Carbonate Biomass Contributions
Source: PLoS One. 2015 Jul 17;10(7):e0132534. doi: 10.1371/journal.pone.0132534 (PMC4505869; doi:10.1371/journal.pone.0132534)
Supplement: S2 Table — The CIs were used to determine if the mean number of Cibicides were significantly more or less common by shell sector. CIs were calculated using one-sample t-tests and the t-statistic is reported for each test. Trace type refers to Cibicides ontogenetic stages represented by their bioerosion traces. Trace types range from T1 (initial recruits that etch the shell surface) to T4 (parasitic adults that made complete boreholes in Adamussium valves). (DOCX) [file pone.0132534.s003.docx]

| **Locality** | **Trace Type** | ***t*-statistic** | **Lower 95% CI** | **Upper 95% CI** |
| --- | --- | --- | --- | --- |
| EC | T1 | 5.22 | 15.54 | 38.63 |
| EC | T2 | 3.03 | 6.24 | 40.84 |
| EC | T3 | 4.94 | 5.23 | 13.85 |
| EC | T4 | 2.57 | 0.215 | 2.99 |
| BOS | T1 | 4.83 | 9.95 | 26.96 |
| BOS | T2 | 5.53 | 32.30 | 75.87 |
| BOS | T3 | 4.63 | 4.20 | 11.98 |
| BOS | T4 | 2.20 | -0.01 | 1.64 |

Explorers Cove (EC); Bay of Sails (BOS);
